# Evaluation of Digital PCR for Absolute RNA Quantification

**DOI:** 10.1371/journal.pone.0075296

**Published:** 2013-09-20

**Authors:** Rebecca Sanders, Deborah J. Mason, Carole A. Foy, Jim F. Huggett

**Affiliations:** 1 Molecular and Cell Biology, LGC, Teddington, United Kingdom; 2 Cardiff School of BioSciences, The Sir Martin Evans Building, Cardiff, United Kingdom; University of Massachusetts Medical, United States of America

## Abstract

Gene expression measurements detailing mRNA quantities are widely employed in molecular biology and are increasingly important in diagnostic fields. Reverse transcription (RT), necessary for generating complementary DNA, can be both inefficient and imprecise, but remains a quintessential RNA analysis tool using qPCR. This study developed a Transcriptomic Calibration Material and assessed the RT reaction using digital (d)PCR for RNA measurement. While many studies characterise dPCR capabilities for DNA quantification, less work has been performed investigating similar parameters using RT-dPCR for RNA analysis. RT-dPCR measurement using three, one-step RT-qPCR kits was evaluated using single and multiplex formats when measuring endogenous and synthetic RNAs. The best performing kit was compared to UV quantification and sensitivity and technical reproducibility investigated. Our results demonstrate assay and kit dependent RT-dPCR measurements differed significantly compared to UV quantification. Different values were reported by different kits for each target, despite evaluation of identical samples using the same instrument. RT-dPCR did not display the strong inter-assay agreement previously described when analysing DNA. This study demonstrates that, as with DNA measurement, RT-dPCR is capable of accurate quantification of low copy RNA targets, but the results are both kit and target dependent supporting the need for calibration controls.

## Introduction

Measuring RNA by reverse transcription real-time quantitative PCR (RT-qPCR) is an established approach for investigating gene expression and viral diagnostics. It is well known that the RT step, required to convert RNA to complementary DNA (cDNA), is imprecise and that different reverse transcriptase enzymes (RT*ase*) can work with considerably different efficiencies [1]. Many of the issues associated with differing RT*ase* efficiencies may be sidestepped by taking advantage of the linear nature of RT and performing relative quantification, with the results expressed as fold changes, or by comparing to a standard curve that is equally affected by the limitations of the RT.

Digital (d)PCR is continuing to gain recognition in the field as an extremely precise and reproducible methodology offering the potential for accurate, robust and highly sensitive measurement without the need for a standard curve [2]. Much work has already been done to meticulously evaluate this technique for DNA molecular measurement [3], [4], [5], [6], [7], [8]. However, a comprehensive evaluation is yet to be established for RNA. dPCR expands the long established premise of molecular quantification by qPCR through facilitating measurement of individual target molecules. Molecules are isolated by limiting dilution and partitioning, before being individually amplified by PCR [7], [9]. Each reaction is then analysed separately. A count of positive partitions may then be used to calculate, using Poisson statistics, an absolute count of target molecules present in the sample [10]. As a result, the need for a calibration curve to assign a value is argued to be unnecessary [4], [5], [7], [11], [12], [13], and this fact has quickly led to the notion that dPCR is calibration free [2]. dPCR may also offer the potential to maximise the accuracy, sensitivity and reproducibility of RNA measurements, for capabilities such as diagnostic mRNA profiling, biomarker analysis and monitoring of viral load.

While this may be true, many studies have demonstrated that the variability inherent in the RT component of the process far outweighs that observed from the PCR step when performing qPCR [14], [15], [16]. Quantification sensitivity differences reported between one-step and two-step RT-qPCR for low copy targets or low concentration samples such as single cells [17], [18], [19], [20], may in part be attributed to gene-specific priming in one-step protocols (as opposed to random hexamers or oligo (dT) commonly used in two-step protocols). An additional consideration when performing RT-dPCR is sample partitioning. For two-step protocols, the cDNA is produced before sample partitioning for dPCR. This therefore must rely on the assumption that the RT step is linear and so the number of cDNA molecules accurately represents the initial number of target RNA molecules. If this is not the case, significant bias may be introduced. Alternatively for one-step protocols, the RNA population is partitioned prior to RT and as such, one RNA target molecule is represented by one positive partition (pending successful amplification). One-step RT-dPCR protocols therefore reduce the potential for bias in this capacity.

In this study we investigated how this characteristic of the RT might affect cDNA production and ultimately influence the dPCR measurement by performing RNA analysis by RT-dPCR and assessing the repeatability, linearity and sensitivity of dPCR measurement. We prepared a Transcriptomic Calibration Material (TCM) and measured both synthetic and endogenous targets, comparing RT-dPCR analysis to UV, and evaluated how different assays and commercially available one-step RT-qPCR kits perform using both endogenous targets and synthetic process controls.

## Materials and Methods

LoBind® tubes were employed throughout this study (Eppendorf, Cambridge, UK). Primer and probe sequences for dPCR were designed in-house using Primer Express, software version 3 (Life Technologies, Paisley, UK) and obtained from Sigma (Dorset, UK). Primers/assays were positioned across different RNA secondary structure motifs (predicted using MFOLD [21], [22], representing both tightly folded and more open regions, depending on the target (Figure S1). Sequences, gene accession numbers and assay concentrations are outlined in Table S1. ERCC RNA concentration and copy number estimates are summarised in Table S2. Assay positions within the respective transcripts are detailed in Table S3. Total yeast RNA [from bakers yeast (*Saccharomyces cerevisiae*), Sigma] at 25 ng/reaction was used as carrier in this study. All samples were diluted using RNA Storage Solution, RSS (Life Technologies), unless otherwise stated.

### Synthetic RNA Transcripts

Six synthetic (ERCC developed targets; External RNA Control Consortium) RNA transcripts (ERCC-00013, −00025, −00042, −00099, −00113, and −00171) were selected for investigation (supplied in plasmid DNA format, courtesy of Dr Marc Salit, NIST, USA). For brevity, the ERCCs shall be subsequently identified without the preceding zeros. Concentrations of plasmid were assigned by the supplier using UV spectrophotometry and converted to copy number using published methods [23]. Copy number conversions were performed using the appropriate extinction coefficient values for dsDNA (50 ng-cm/µL) or RNA (40 ng-cm/µL).

ERCC RNA was prepared from the corresponding plasmid DNA, as described previously [24] *In vitro* transcription (IVT) performed using MEGAscript® T7 Kit (Life Technologies): 37°C overnight incubation, with Turbo DN*ase* treatment. IVT ERCC RNA concentrations and insert sizes were subsequently estimated using Nanodrop UV spectrophotometry (Thermo Scientific**,** Massachusetts, USA) and 2100 Bioanalyzer (Agilent, West Lothian, United Kingdom), respectively. 2100 Bioanalyzer data may be found in Figure S2. Samples were diluted to approximately 1 ng/µL in RSS and aliquots stored at −80°C. Concentrations and copy number estimates are reported in Table S2.

### Cell Lines: Endogenous Targets

Three human cell lines were employed for production of complex background material for endogenous target selection; Hep-G2 (organ: liver, disease: hepatocellular carcinoma), SaOS-2 (organ: bone, disease: osteosarcoma) and Hs 683 (organ: brain, disease: glioma), (all cell lines from ATCC, Teddington, UK). Culturing details given in Appendix S1.

Based on confluency and cell size, eight to fourteen flasks were prepared for each cell type, as outlined in Appendix S1. Medium was removed, cells washed in Hanks Balanced Salt Solution (HBSS; PAA Laboratories, Somerset, UK) and TRIzol (Sigma) added directly to cell monolayers (17.5 mL per T-175 flask) and passed three times over the entire surface of the flask to ensure cell lysis. Lysates were transferred to 50 mL round-bottomed Falcon tubes and stored at −80°C until RNA extraction. Replicate T-175 flasks (one per cell line used) generated at the same time and under the same conditions were used for cell enumeration and viability estimates using a Vi-Cell (Beckman Coulter, High Wycombe, UK). For cell counting, cells were detached post HBSS wash using 5 mL Trypsin/EDTA (Sigma) for 5 min at 37°C and neutralised with 5 mL culture medium before Vi-Cell analysis.

Total RNA was extracted from cell lysates by following the standard TRIzol protocol (Invitrogen), details given in Appendix S1. Total RNA solutions were then treated with recombinant (r)DN*ase* I (Life Technologies), as per manufacturer’s protocol (1 U rDN*ase* I reagent added per 4 µg of total RNA, incubated at 37°C for 30 min). These preparations were then purified using RNeasy midi kit (Qiagen), assessed for quantity (yield; Nanodrop), and subsequently pooled (per cell-type).

Following total RNA extraction, DN*ase* treatment and clean-up, pooled cell-line RNA samples were subjected to standard quality metrics for concentration and integrity (Nanodrop and 2100 Bioanalyzer, respectively). Neat samples (resuspended following clean-up in nuclease-free water, between 110–700 ng/µL) were then stored in aliquots at −80°C.

### Preparation of Transcriptomic Calibration Material

Pooled cell line RNA stocks were diluted in RSS to 250 ng/µL (Hep-G2 and Hs683) or 100 ng/µL (SaOS-2), and the complex background material prepared by mixing different proportions of each cell line RNA to a final concentration of 50 ng/µL (Proportions: 0.755 Hep-G2, 0.205 Hs 683, 0.04 SaOS-2). A mix containing all six ERCC transcripts was spiked into the mixed ratio cell line solution, at approximately 1.0E+06 copies/µL (final concentration), to produce the Transcriptomic Calibration Material (TCM) for analysis. The TCM solution was aliquoted (150 µL) to generate 245 replicate units prior to storage at −80°C.

### RT-dPCR Analysis

dPCR experiments were performed using the Fluidigm Biomark platform. Both 12.765 and 48.770 chip formats were utilised. Assays were first optimised using the Prism 7900 HT real-time PCR system (Life Technologies) before transfer to the Biomark. One-step RT-dPCR utilised AgPath-ID one-step RT-PCR reagents (Ambion). Master reactions comprised RT-PCR buffer/master mix (1×), RT enzyme (1×), GE sample loading reagent (1×, Fluidigm), sequence-specific gene assay (Table S1. RT priming was gene-specific due to one-step process), 25 ng/reaction Yeast total RNA carrier and RNA at various concentrations (Table 1). These master reactions were added to dPCR chip inlets, a proportion of which is loaded per panel (see Appendix S2 for volumetric details). Samples were analysed in triplicate (kit comparison) or replicates of six panels (Quantification Sensitivity experiment). Reaction mix (i.e. master mix, gene specific assay and RNA) was loaded into sample inlets and delivered to nanolitre partitions by an integrated fluidic circuit controller. Thermal cycling conditions: (RT) 45°C for 30 min, (RT*ase* inactivate/denature) 95°C for 15 min, (PCR) 40 cycles 95°C for 15 s and 60°C for 60 s. Analysis was performed utilising dPCR analysis software (Fluidigm), version 3.0.2. dPCR calculations are explained in further detail in the Appendix S2. Adherence to the MIQE guidelines is detailed in Table S4.

**Table 1:** Sample dilutions analysed. Derived by UV spectrophotometry (ERCCs only).

Experiment	Assay	RNA target copies per panel*	Replicates
One-Step RT-qPCR Kit Comparison by dPCR	ERCC-25 and ERCC-99	∼1896	1 panel/assay, 3 replicate chips
Comparison between dPCR and UV Measurement	All six ERCCs	∼200–400	3 panels/assay
RT-dPCR Quantification Sensitivity	ERCC-25 and ERCC-99	∼500, 250 100, 50, 25, 10 or 5	6 panels/dilution/assay, 2 replicate chips
Evaluation of Reverse Transcriptases	ERCC-25, ERCC-99, UBC and MMP1	∼1886	3 panels/assay duplex, 2 replicate chips

* Dilutions are quoted based on RNA copies per dPCR panel. RNA concentrations were estimated by UV and converted to copy number using published methods [23]. No template controls (NTCs) for every experiment resulted in no amplified signal observed.

A count of partitions showing positive amplification can be made and an absolute target concentration elucidated. “Estimated copies” or “Copies per panel” refer to the number of targets on the panel following a Poisson correction, to account for the fact that some positive partitions will contain more than one molecule. As the number of positive partitions increases, so does the probability that some partitions will contain more than one target molecule. See Appendix S2 for calculations. Examples of dPCR output are provided in Figure S3.

For one-step kit comparison, two further commercial kits were evaluated; Quantitect Probe one-step RT-PCR Kit (Qiagen) and Superscript III Platinum one-step RT-qPCR system w/ROX (Invitrogen). Both the Ambion (Multiscribe) and Invitrogen (Superscript III) RT*ases* are derived from Moloney murine leukemia virus (MMLV) RT*ase*. Alternatively, the Qiagen (Omniscript and Sensiscript) RT*ases* are derived from a unique source (undisclosed). The Qiagen RT*ases* maintain RN*ase* H activity, while the Ambion and Invitrogen RT*ases* are claimed to have reduced RN*ase* H activity.

### One-Step RT-qPCR Kit Comparison by dPCR

Initially, quantification was assessed for two external (ERCC-25 and ERCC-99) targets in both uniplex and duplex formats, between the three commercial one-step RT-qPCR kits: AgPath ID (Ambion), Quantitect (Qiagen) and Superscript III (Invitrogen). RT-dPCR was performed using Fluidigm Biomark 12.765 dPCR chips, n = 1 panel, plus three replicate experiments. Sample was diluted to approximately 1896 copies per panel (or 2062 copies/µL added to master mix), based on UV estimates. Following this, ERCC-25 and ERCC-99, plus two endogenous (UBC and MMP1) targets were compared between the kits. These assays were analysed in duplex: ERCC-25 with ERCC-99 (duplex A), UBC with MMP1 (duplex B), and ERCC-25 with UBC (duplex C). Sample was diluted to approximately 1886 copies per panel (or 1640 copies/µL added to master mix, for ERCC targets), based on UV estimates. RT-dPCR was performed using Fluidigm Biomark 12.765 dPCR chips, n = 3 replicate panels, plus two replicate experiments.

### Comparison between dPCR and UV Measurement

Measurement variability of six ERCC targets was tested using RT-dPCR evaluated as above (AgPath ID kit, Ambion). ERCC targets were spiked into cell line-derived total RNA at approximately 1.0E+06 copies/µL (estimated by UV), enabling evaluation of potential assay bias. Sample was diluted to approximately 200–400 copies per panel. RT-dPCR was performed using Fluidigm Biomark 48.770 dPCR chips, n = 3 replicate experiments. Assays were analysed in uniplex.

### RT-dPCR Quantification Sensitivity

An evaluation of RT-dPCR quantification sensitivity was performed using ERCC-25 and ERCC-99 assays. Based on UV estimated values, sample was diluted in 0.5% Tween 20 (Sigma) to approximately 500, 250, 100, 50, 25, 10 and 5 copies per panel (equivalent to 3077, 1538, 615, 308, 154, 62 and 18 copies/µL, respectively). Volumetric dilutions were performed independently for each dilution, rather than sequentially, to avoid volumetric error propagation during dilution steps. RT-dPCR was performed using Fluidigm Biomark 48.770 dPCR chips, n = 6 panels per dilution, plus three replicate experiments. Assays were analysed in duplex.

### Statistical Methods

All statistical analyses were performed using MS Excel 2007 and the R statistical programming environment (http://www.r-project.org/). All data sets incorporated ANOVA calculations.

#### One-Step RT-qPCR kit comparison by dPCR

The square of the copy numbers was needed in order to stabilise the difference in variance between groups. Standard uncertainties have 2 degrees of freedom, converted to expanded uncertainty with coverage factor (k) = 4.3.

#### Comparison between dPCR and UV measurement

Weighted regression was used to stabilise the different variance between groups. Standard uncertainty estimates were made to 3 significant figures and have 2 degrees of freedom, with k = 4.3 to convert to expanded uncertainties. Only dispersion due to plate-to-plate variation was included. dPCR plate-to-plate variability was estimated by pooling the data for all six ERCCs. The relative standard deviation was approximately 7.59% (46,000 copies) with 12 degrees of freedom (18 data points minus the six estimated group means).

#### RT-dPCR quantification sensitivity

A linear mixed model fit was used with experiment as random effect. Additionally, an ANOVA was applied removing experiment from the model and applying a classical fixed effect linear model fit (with only assay and dilution as factors).

#### Endogenous versus synthetic targets

The analysis was split into four groups, one per assay. The square root of the copy numbers was needed in order to stabilise the difference in variance between groups. The Qiagen kit always resulted in zero positive partitions for MMP1, which was therefore removed from the data set.

## Results

### One-Step RT-qPCR Kit Comparison by dPCR

**Figure 1:**
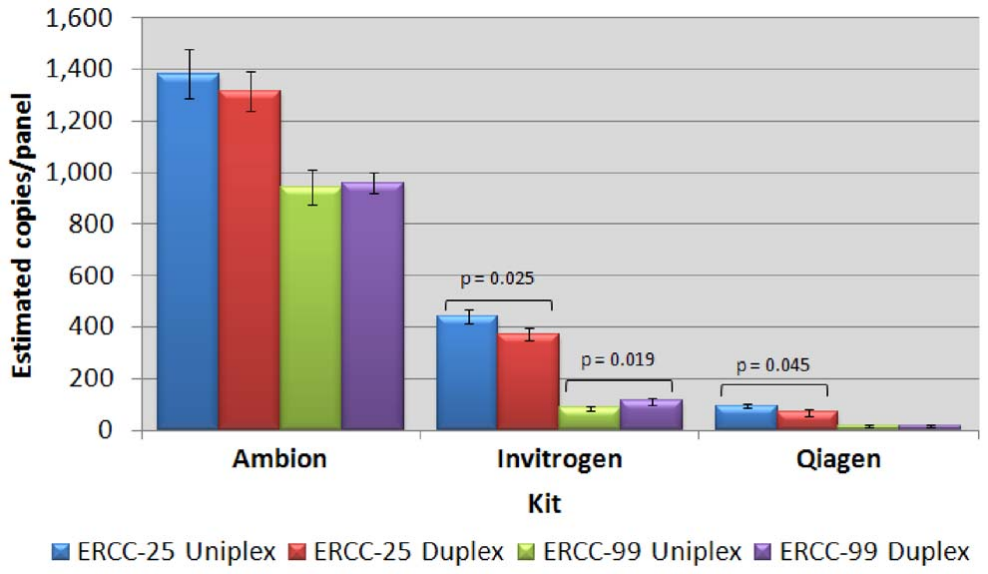
One-step kit comparison. Three different one-step RT-qPCR kits were compared in both uniplex and duplex formats, by dPCR. Two external targets, ERCC-25 and ERCC-99 were analysed. Error bars: 95% Confidence intervals. n = 3 replicate panels. Equivalent UV estimates: ERCC-25 1185 copies/panel, 95% CI 17.34. ERCC-99 1185 copies/panel, 95% CI 26.19.

Three commercially available kits were compared for quantitative performance by RT-dPCR. The three kits were initially assessed using both uniplex and duplex formats for quantification of two synthetic RNA targets: ERCC-25 and ERCC-99 (Figure 1). The choice of kit significantly affected RNA quantification (p<0.0001) with the Ambion kit consistently yielding the highest signal. A significant difference was also observed between uniplex and duplex formats for the Qiagen (ERCC-25 p = 0.045) and Invitrogen (ERCC-25 p = 0.025, ERCC-99 p = 0.019) kits but not the one supplied by Ambion (ERCC-25 p = 0.347, ERCC-99 p = 0.736), (Qiagen ERCC-99 p = 1.000); however this difference was considerably smaller than the inter kit differences (Figure 1).

Consistent ratios for ERCC-25:ERCC-99 between uniplex and duplex measurements were not maintained between kits suggesting an assay-dependent as well as a kit associated difference (Table 2). The ERCC-99 assay consistently resulted in lower estimated copies than that for ERCC-25 (with all kits), despite being added at the same concentration; as estimated by UV.

**Table 2:** Three one-step kit comparison with uniplex and duplex formats.

Method	Format	ERCC-	Positive Partitions	Copies per panel*	Ratio†	Standard Uncertainty
Ambion	Duplex	25	627	1316	1.37	0.051
		99	546	959		
	Uniplex	25	639	1383	1.47	0.076
		99	541	944		
Invitrogen	Duplex	25	295	373	3.31	0.223
		99	104	113		
	Uniplex	25	335	442	5.18	0.262
		99	81	85		
Qiagen	Duplex	25	68	71	4.22	0.588
		99	17	17		
	Uniplex	25	89	95	5.57	0.906
		99	17	17		

* Copies per panel calculated from the number of positive partitions using the Poisson correction.

† Ratio of ERCC-25/ERCC-99 dPCR values with standard uncertainties. Ratios calculated using copies per panel. Standard uncertainty calculated by dividing the standard deviation by the square root of n (number of replicate measurements).

### Comparison Between dPCR and UV Measurement

**Figure 2:**
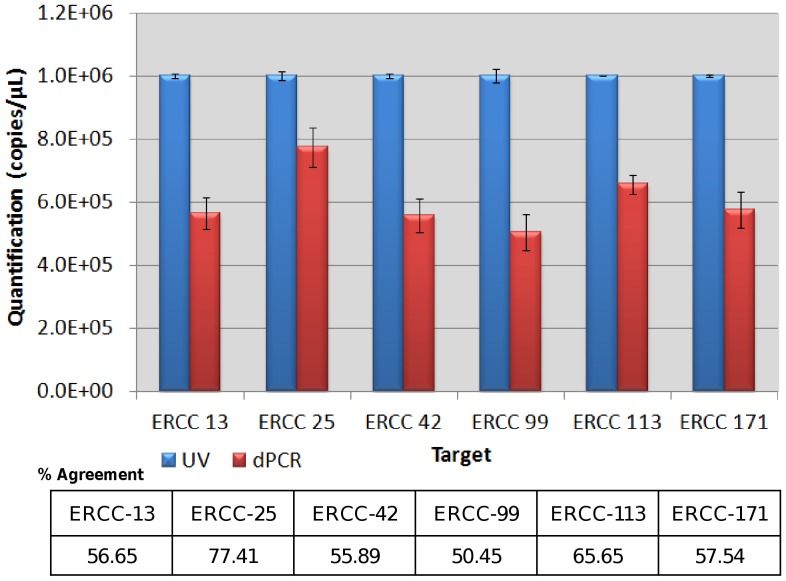
dPCR versus UV quantification. Six external targets (ERCC-13, −25, −42, −99, −113 and −171) were assessed by both one-step dPCR, utilising the Ambion one-step RT-qPCR kit, and UV measurement. Error bars: 95% Confidence intervals. n = 3 replicate dPCR experiments or UV measurements.

To investigate this disparity further, RT-dPCR measurements using the Ambion kit were compared when measuring a further four ERCC targets (all six present within the TCM) (Figure 2). dPCR estimates of ERCC transcript quantities were on average 40% lower than when measured by UV (p<0.0001). Bioanalyzer quantification for all six synthetic targets was comparable to nanodrop concentration estimates (p = 0.660, with an average difference between the two approaches of 1.02). The differences observed in absolute quantification between dPCR and UV were assay-specific (Figure 2). The number of dPCR positive partitions for ERCC-25 was closest to UV at 77.41% agreement, whereas ERCC-99 displayed the lowest agreement at 50.45%, as previously described. Furthermore, there was no inter-plate difference observed despite 5–6 days between runs.

### Linearity and Sensitivity of RT-dPCR

**Figure 3:**
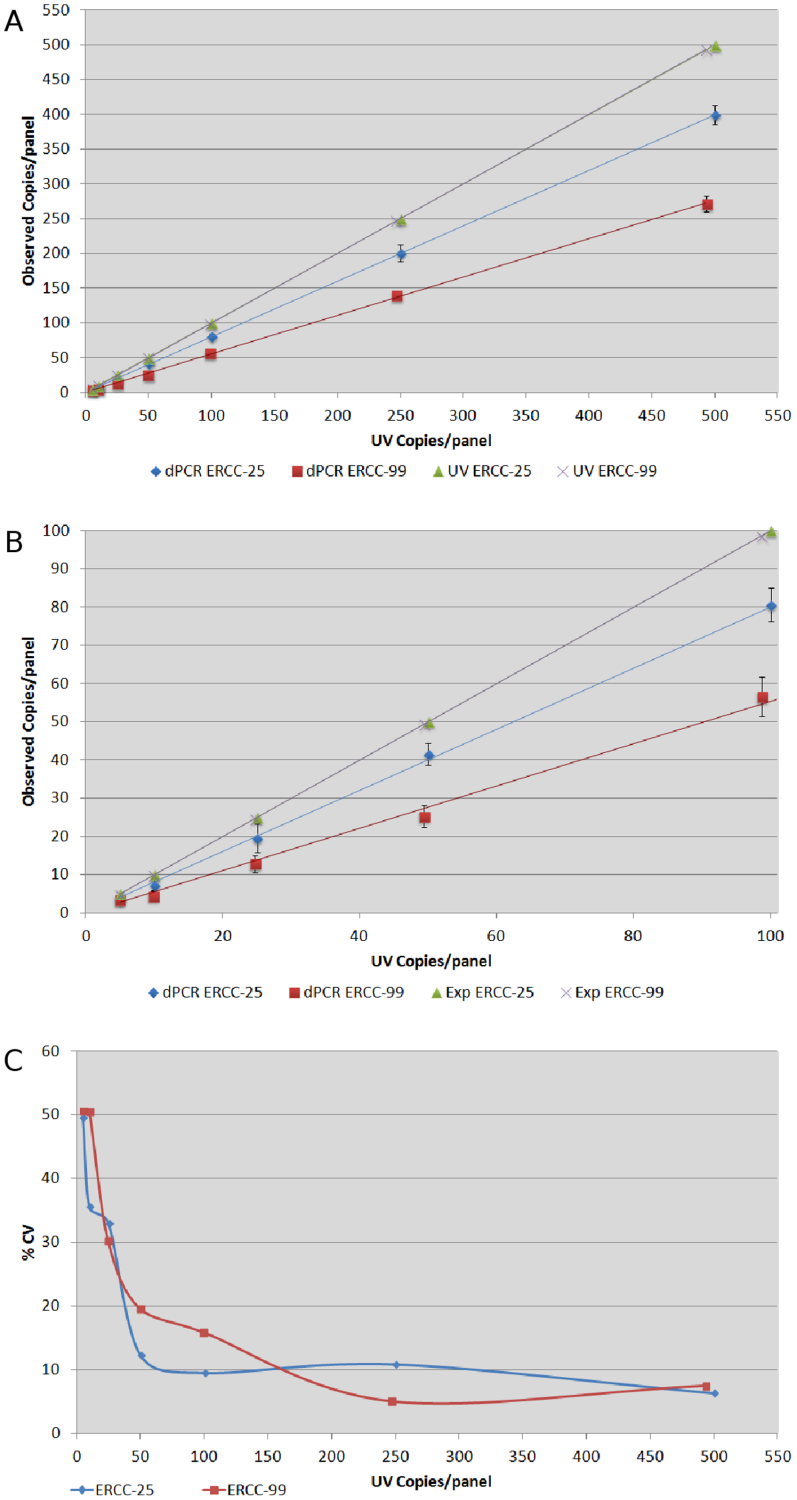
dPCR sensitivity for RNA measurement. Assessment of RT-dPCR quantification sensitivity, using independent dilutions and quantifying ERCC-25 and ERCC-99 external targets in a duplex format. n = 6 panels per dilution, plus two replicate experiments. UV data based on initial UV quantification of stock and predicted target levels following volumetric dilutions. (A) & (B) dPCR sensitivity. (B) Focus on lowest level target dilutions. Error bars: 95% Confidence intervals. (C) Precision of dPCR quantification compared to UV.

An additional aim was to identify RT-dPCR sensitivity and linearity of measurement for low copy targets. This was performed utilising the Ambion kit alone, due to its superior capabilities throughout our initial analyses. A dilution series of two synthetic RNA targets, ERCC-25 and ERCC-99, were analysed in duplex (Figure 3). Dilutions were performed based on UV evaluation, using dH_2_O 0.5% v/v Tween 20 as diluent, to generate samples equating to approximately 500, 250, 100, 50, 25, 10 or 5 copies/panel.

There was a significant difference identified between the two targets agreement with UV values, p<0.0001 (Figure 3A & B), which concurred with our previous observations (Figure 2). Both ERCC-25 and ERCC-99 displayed linear quantification capabilities, with good precision (CVs of less than 10%) achievable down to 50 UV assigned copies (Figure 3C).

### Further Evaluation of Reverse Transcriptase’s Targeting Endogenous Transcripts

**Figure 4:**
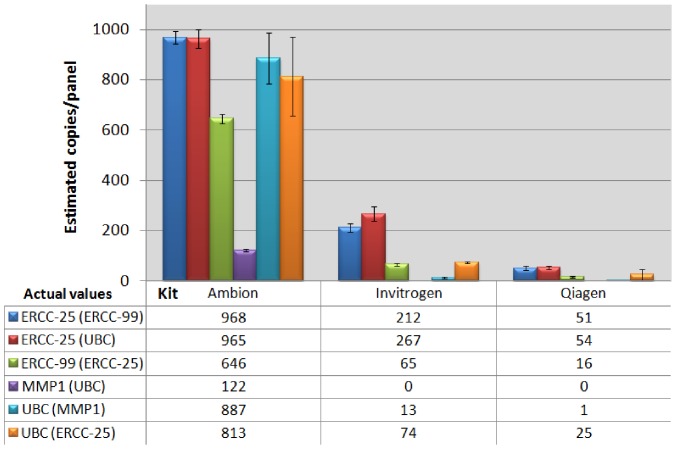
Evaluation of Reverse Transcriptases. Three different one-step RT-qPCR kits were compared in different duplex formats, by dPCR. Quantification for external (ERCC-25 and ERCC-99) and endogenous (MMP1 and UBC) targets was evaluated. ERCC-25 with ERCC-99 (duplex A), UBC with MMP1 (duplex B), and ERCC-25 with UBC (duplex C). In the key/tabulated values, the assay in brackets is the duplex partner for the assay whose positive partition values are being displayed. Error bars: 95% Confidence intervals. n = 3 replicate panels, plus two replicate experiments.

In order to investigate the applicability of our findings to real samples, the same three, one-step RT-qPCR kits were tested to compare measurement of endogenous targets alongside external controls in various duplex combinations in the TCM (Figure 4). Again for each target, there was a significant effect of kit on dPCR quantification (all p values <0.0001). For endogenous targets, the Ambion kit yielded the highest quantification values, as previously observed with external controls: although the variability observed for UBC was higher.

To establish whether different plex pairings influenced RT-dPCR results, duplex reactions were performed pairing different targets (Duplex A: ERCC-25+ ERCC-99. Duplex B: MMP1+ UBC. Duplex C: ERCC-25+ UBC). As observed above, there was a significant difference between the kits, but no significant difference observed in dPCR values between ERCC-25 or UBC when assessed in different duplex reactions using the Ambion reagents (ABC), p = 0.061 and 0.92, respectively. Therefore, for these targets, assays did not influence the quantification result of their duplex partners.

## Discussion

In this study we used a Transcriptomic Calibration Material (TCM) containing synthetic RNA transcripts in a complex background made of mixtures of human cell line total RNA. This was used to both evaluate dPCR measurement and demonstrate the applicability of the TCM for supporting accurate RNA enumeration by RT-dPCR.

The findings from the one-step kit comparison by dPCR (Figure 1, Table 2) indicate that there can be large numbers of RNA molecules present within the dPCR partitions that are not being detected with dPCR because either they are not converted to cDNA or are being converted to cDNA but not being amplified by the PCR: and that this is kit and/or transcript dependent. Furthermore, the UV measurement may potentially overestimate the initial valuation. This is explored in more detail below.

The analysis method was shown to significantly affect the RNA quantification result. There may be a number of reasons explaining the significant difference observed between dPCR and UV methodologies. While dPCR makes an absolute count of specific amplified cDNA target molecules, UV cannot discriminate between nucleic acid species, non-target RNA and fragmented/degraded/non-amplifiable targets [7], [25], [26], [27], [28], [29]. This could contribute to the consistent increased RNA concentration estimated by UV. However, the concordance between UV and the 2100 Bioanalyzer suggest these additional factors are not playing a major role. Another explanation for the discrepancy is that the RT-dPCR measurement value may be underestimating the true concentration. Quantification of RNA reflects only the number of target cDNA molecules converted from the original RNA. This may or may not give an accurate estimate for the original concentration of the RNA molecules of interest [30]. Not only have we shown here the potential for RT sensitivity and variability to affect dPCR estimation, as previously reported when using qPCR [1], [31], but our previous studies have shown similar disparity between dPCR and UV valuation when measuring DNA targets [7], suggesting the PCR step in the RT-dPCR may also contribute to the observed differences.

Our linearity and sensitivity data clearly show a pattern of increased variability with the increase of dilution factor below 50–100 estimated copies. We have previously demonstrated that when analysing DNA targets, dPCR is highly precise down to 16 copies/panel [7] suggesting RNA measurement is more variable.

The magnitude of the quantification difference between kits was not consistent between different targets, both synthetic and endogenous, suggesting an additional assay specific and kit associated bias. There was a greater difference between kits when measuring endogenous targets than for synthetic targets. Furthermore, both Invitrogen (1 positive partition) and Qiagen (0 positive partitions) kits were unable to provide satisfactory quantification values for MMP1 despite being measured with six replicates totalling some 4590 reactions. However, as the Ambion kit only measured on average 112 MMP1 positive partitions, it would suggest that this transcript was below the limit of detection for the two former kits. Measurement, or specifically enzyme, efficiency/sensitivity is an important consideration when measuring low abundance RNA targets, in order to avoid false negative results and our data suggests that choice of kit is crucial for ensuring the most sensitive result when performing RT-dPCR. It should also be noted that while MMP1 target was present at low abundance in the dilutions tested, evaluation of a more concentrated sample may circumvent the sensitivity issues associated with the two kits. Therefore, this must also be considered when validating protocols, and where possible, low copy measurements should be avoided.

### Causes of Differing RT-dPCR Results

One of the most striking findings of this study is the large inter assay and inter kit difference in the estimated copies for a given target. There are a number of potential causes for these observations. It is clear from our data that some, if not all, of the kits analysed during this study were not measuring all the RNA molecules that were present. There may be a number of different reasons for this. The assumption that DNA measurement by dPCR can be precise, reproducible, and absolute cannot be readily extrapolated to the measurement of RNA [30]. The RT step introduces an additional source of variability. It is a well-documented fact that RT does not convert all RNA to cDNA [32], [33]. RT inefficiency and variability may account for the majority of measurement divergence, especially given that qPCR has been shown to be extremely sensitive and efficient [34], [35]. In addition, several studies have shown that RT reaction components may have a reversible inhibitory effect on the subsequent qPCR, the magnitude of which depends on the RT system [17], [27], [36], [37], [38]. While it would be hoped that in the one-step kits investigated in this study the RT components would have minimal effect on the PCR step, one cannot rule out the possibility that as well as RNA not being converted to cDNA, failed subsequent amplification of the cDNA may also explain the underestimation.

In our recent study, we documented a dPCR phenomenon termed molecular dropout [39]. This event is characterised as a failure to detect the presence of a target molecule during dPCR. In other words, the target molecule is present in the partition but is not amplified. Given this precedent, it is therefore plausible to assume that molecular dropout, either at the cDNA or RNA stage of the RT-dPCR process, on a much larger scale to that measured by dPCR alone, may partly explain our findings. Moreover, it is possible that different enzymes may be affected to different degrees by this phenomenon. Several factors may contribute to molecular dropout including assay sensitivity, reagent inhomogeneity, template complexity and matrix effects (e.g. inhibition).

Template secondary structure and position of the assay is known to impact on the RT-qPCR reaction [34] and may contribute to this molecular dropout. The potential impact of template secondary structure was assessed [21], [22] to evaluate whether this could be a cause for molecular drop out and determine positional influences contributing to assay performance. All templates displayed a degree of secondary structure within the amplicon region (Figure S1). When concentrating on the regions complementary to the reverse primer (used in the RT to prime cDNA synthesis), all templates exhibited some degree of stem-loop structures. However, the 3′ ends of the reverse primer complementary region showed differing secondary structures. For example the 3′ end within ERCC-25 was within an open (loop) structure while for ERCC-99, the final base was designed to bind to a closed (stem) region (Figure S1 D & F). Given that the primers are extended from the 3′ end, this may explain why ERCC-25 consistently gave a higher value than ERCC-99 despite their being present at the same copy number. The assay-specific bias observed between kits for different external and endogenous targets maybe in part explained by predicted template secondary structures and this would also appear to be kit specific.

The recommendation from the MIQE guidelines [34] that RT primers be designed to stem loops to improve qPCR maybe a particularly important consideration when performing RT-dPCR to improve assay sensitivity. Further work is required to build on the hypothesis that RNA structure will effect RT-dPCR sensitivity, but our findings suggest reaction efficiency may in part reflect the ability of an enzyme to negotiate strong secondary structures and successfully progress the course of the reaction and that this is specific to different kits.

There may be other factors contributing to RT yields. For example, the samples used were sourced from cell line lysates. Co-extracted inhibitors may affect different reverse transcriptases to different degrees. Furthermore, components of total RNA, such as rRNA and tRNA may additionally inhibit RT*ase* efficiency [40], by competing for reagents and producing undesired products. However, the manufacturers claim that the RT*ase* used in the Invitrogen kit is not significantly inhibited by such total RNA components. These considerations taken together may in part explain the disparity displayed between different one-step RT-qPCR kits.

As may be seen from this comparison, despite the accuracy conferred by dPCR, analysis of RNA using RT-dPCR needs to be approached with caution. While for RNA measurement the precision of the RT-dPCR technique is high, it nonetheless introduces increased variability into the measurement value than dPCR alone [7]. The significant differences observed between kit sensitivities, particularly for low abundance targets (MMP1), highlight the importance of reagent choice and protocol consistency as critical if data sets are to be meaningfully compared. Furthermore, the inability to detect certain targets may be due to the choice of RT*ase*/kit and all experimental plans should therefore be validated appropriately before embarking upon studies analysing important samples.

For accurate RNA analysis by RT-dPCR it is possible that unknown measurements should be properly correlated to an appropriate measurement standard, with a well-defined value and uncertainty [30], [41], [42], [43]. It may also be the case that while, unlike RT-qPCR, RT-dPCR may not need a calibration curve to assign a value, some kind of calibration molecule will be required to compensate for the assay/kit differences observed here. All samples may be normalised to a calibrator sample, also known as a reference sample, in a similar way as performed for relative quantification by RT-qPCR. It is possible that in some cases where assay bias is observed, only gene specific calibrators will be appropriate. For accurate absolute quantification our data suggest use of a calibrant sample, with an accurate assigned value, will allow straightforward correction of dPCR data to account for differences in enzyme efficiencies, inhibitors and molecular dropout. Such dPCR-specific calibrant materials are yet to be developed and approaches combining validated external and endogenous control materials, as described here, represent a possible strategy. The full power of this technique may only be realised on their experimental incorporation.

## Conclusion

This study has shown that dPCR is capable of making precise measurements of synthetic and endogenous RNA molecules in a complex RNA background. RT-dPCR quantification of RNA targets was significantly lower than that derived from UV values suggesting a possible underestimation bias. Furthermore, absolute measurements differed between the three one-step kits assessed, with bias in detection sensitivity. Linearity and precision were sustained for duplex dPCR measurement of synthetic RNA using the Ambion kit, while sensitivities differed between RNA targets. dPCR is unencumbered by the restraints of calibration curve measurements, however, the employment of dPCR-specific calibrant materials (reference samples) would facilitate greater accuracy for absolute quantification. Furthermore, use of the TCM shows the applicability of RT-dPCR for the target-dependent selection of suitable RT enzymes. This study is novel in demonstrating application of RT-dPCR for absolute quantification of RNA endogenous and synthetic targets. Our findings give strong weight to the applicability of RT-dPCR to measurement fields including RNA diagnostics and RNA viral measurement.

## Supporting Information

Figure S1 RNA Secondary Structure Predictions from mFold. (A) MMP1, (B) UBC, (C) ERCC-13, (D) ERCC-25, (E) ERCC-42, (F) ERCC-99, (G) ERCC-113 and (H) ERCC-171. Green highlighted regions indicate amplicon. Folding predictions were performed at 45°C (temperature of RT step).
(DOCX)

Figure S2 Integrity assessment of Synthetic RNA Transcripts. 2100 Bioanalyzer quantification for all six synthetic targets was comparable to nanodrop concentration estimates (p = 0.660, with an average fold change between the two measurements of 1.02).
(DOCX)

Figure S3 Typical dPCR output data from this study. Both amplification plots and heatmaps are shown. Amplification plots display ΔRN versus cycle number. Heatmaps are the corresponding schematic representations of positive partitions as detected by the Biomark instrument. Black = no amplification. Red = FAM amplification. Blue = HEX amplification. Threshold was adjusted to eliminate cross talk between the filters (FAM versus HEX). (A) One-Step RT-qPCR Kit Comparison by dPCR. (B) Endogenous versus Synthetic Targets.
(DOCX)

Table S1 Primer and probe sequences.
(DOCX)

Table S2 ERCC RNA concentration and copy number estimates.
(DOCX)

Table S3 Assay Positions.
(DOCX)

Table S4 MIQE checklist for authors, reviewers and editors.
(DOCX)

Appendix S1 Materials and Methods.
(DOCX)

Appendix S2 dPCR Calculations Explained.
(DOCX)
