# Supplementary figures and images for: Evaluation of Digital PCR for Absolute RNA Quantification
Source: PLoS One. 2013 Sep 20;8(9):e75296. doi: 10.1371/journal.pone.0075296 (PMC3779174; doi:10.1371/journal.pone.0075296)

**Figure S1**


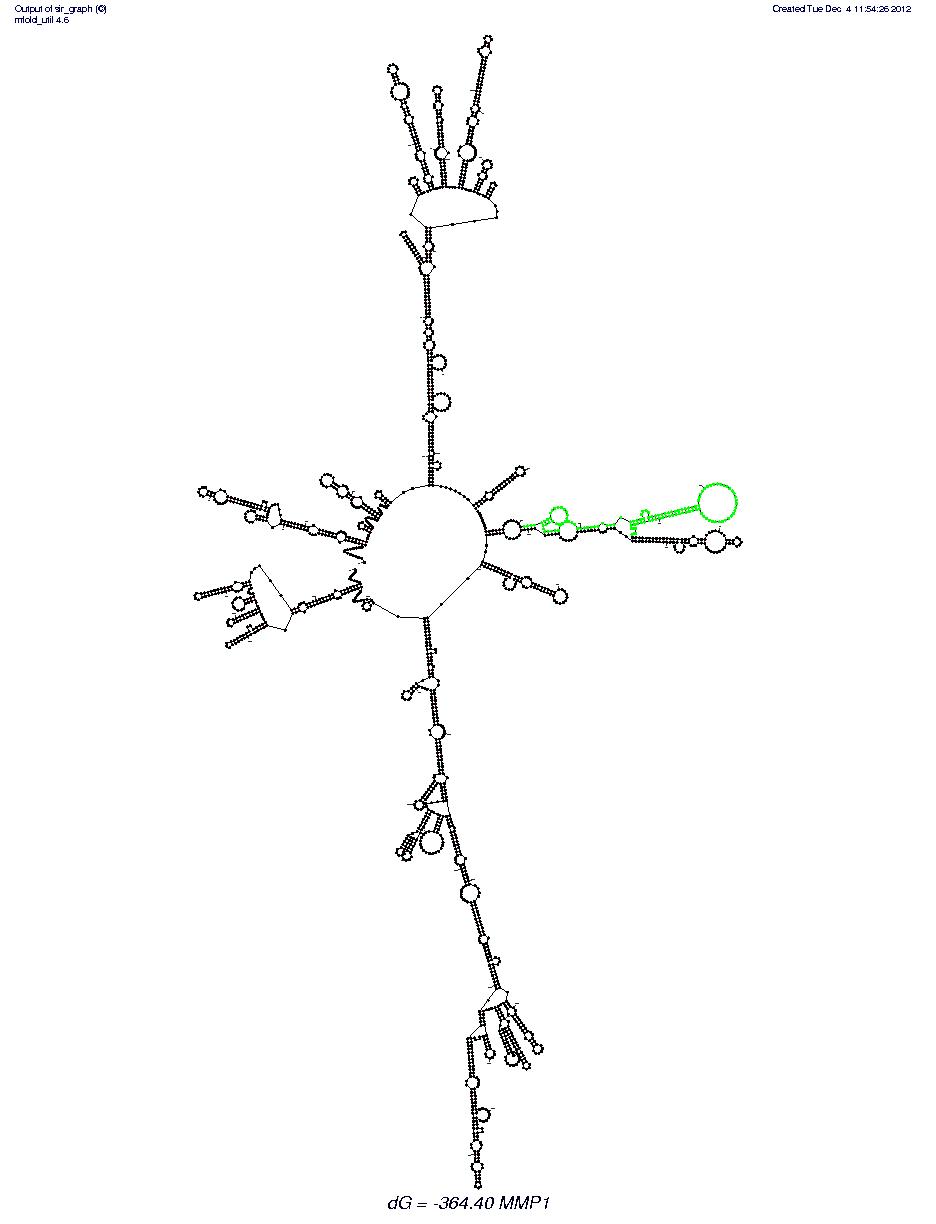


**A**


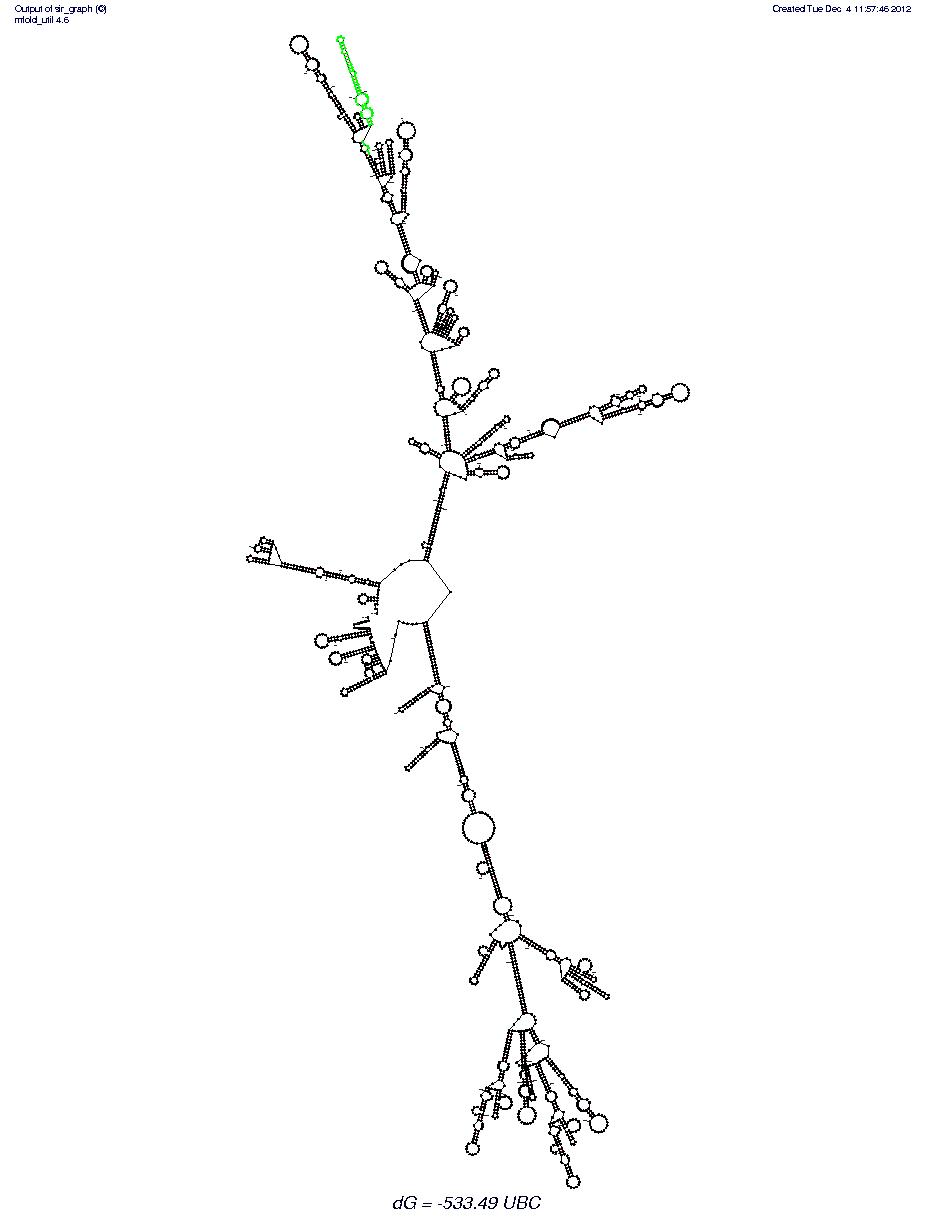


**B**


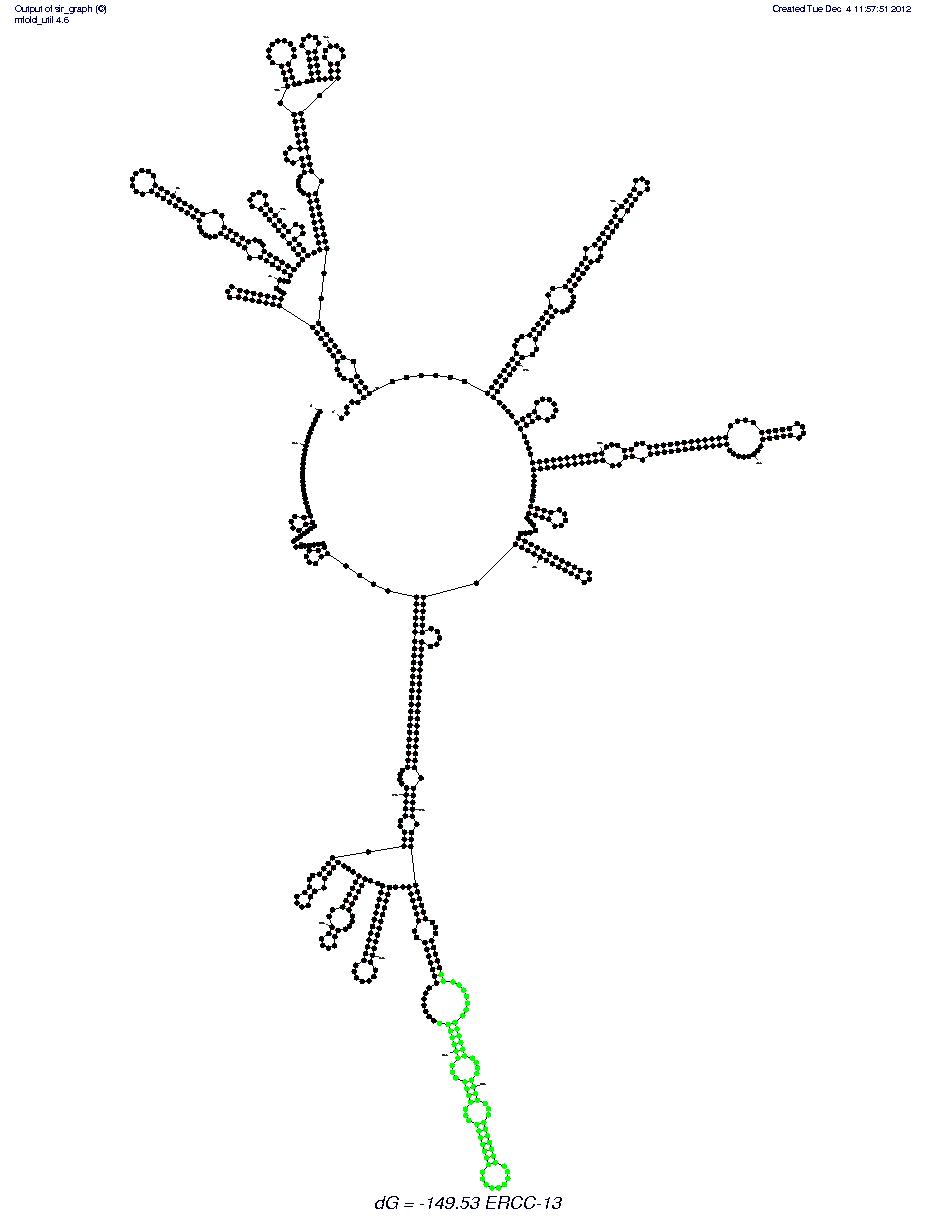


**C**


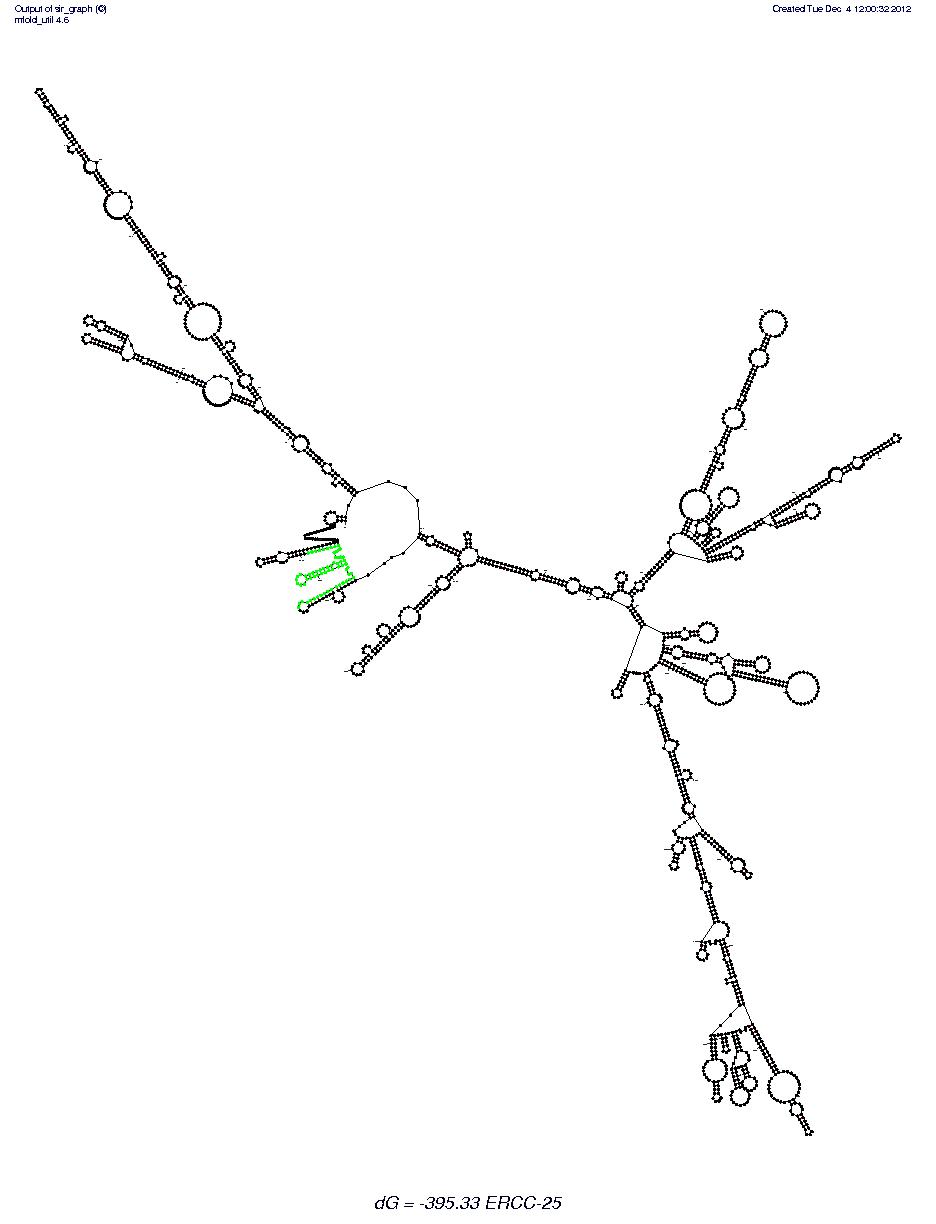


**D**


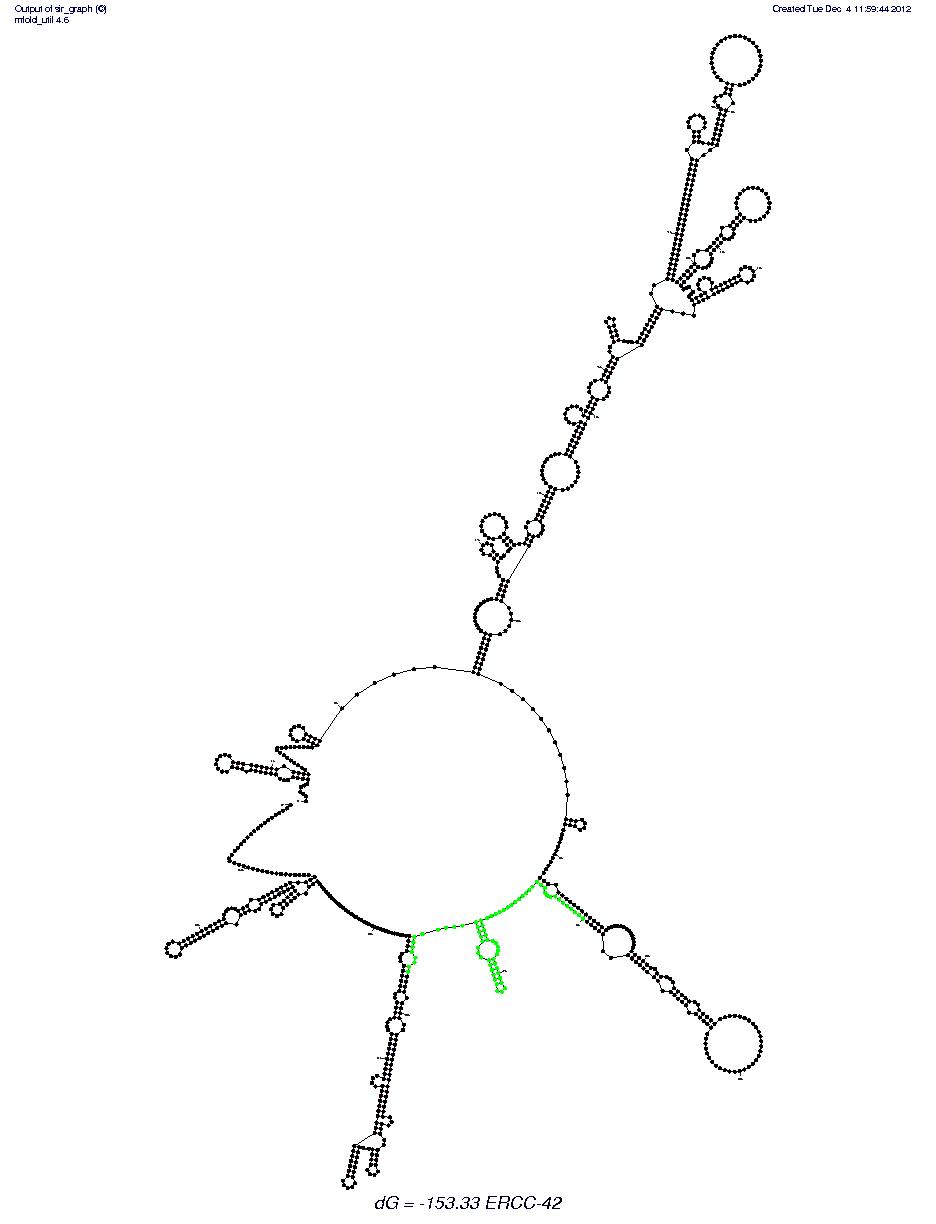


**E**


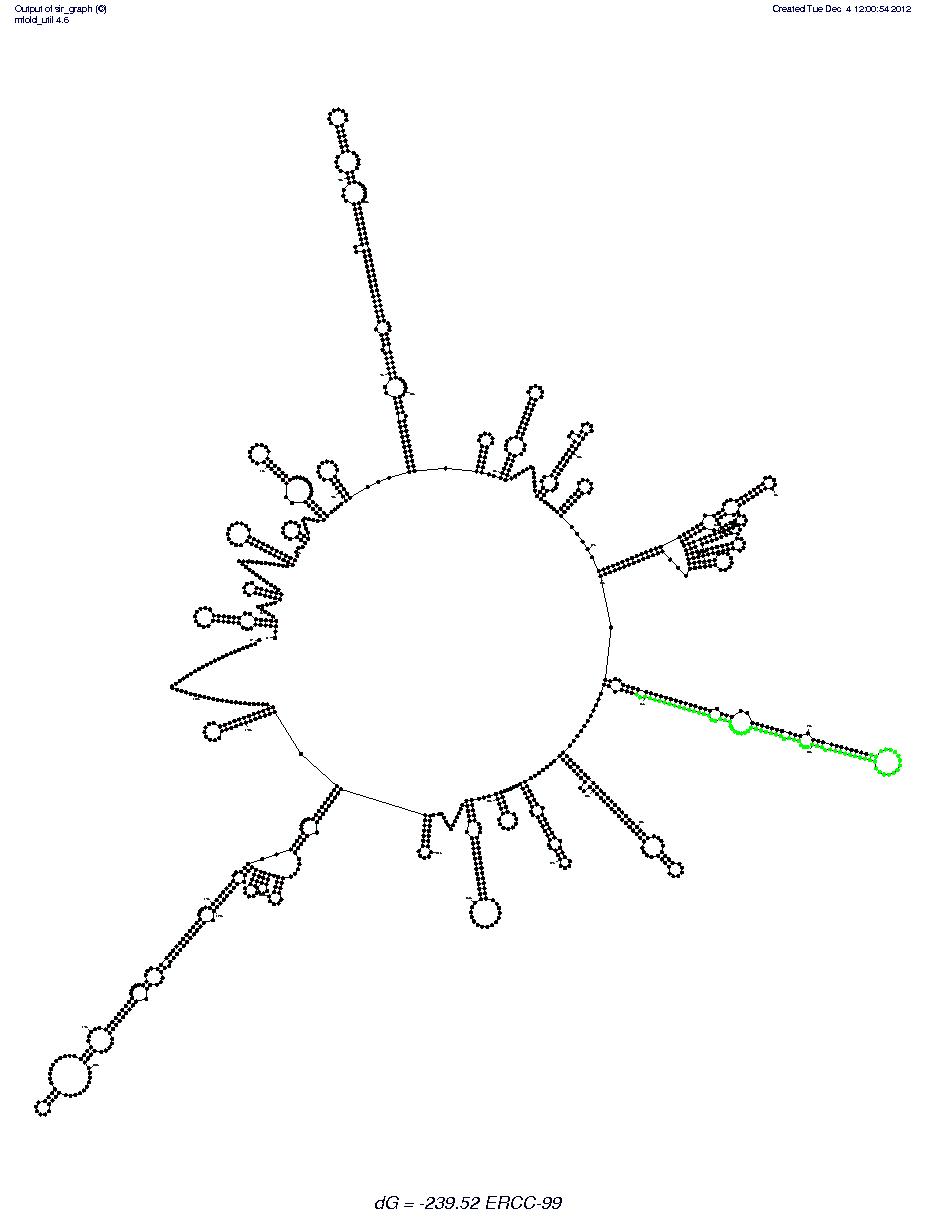


**F**


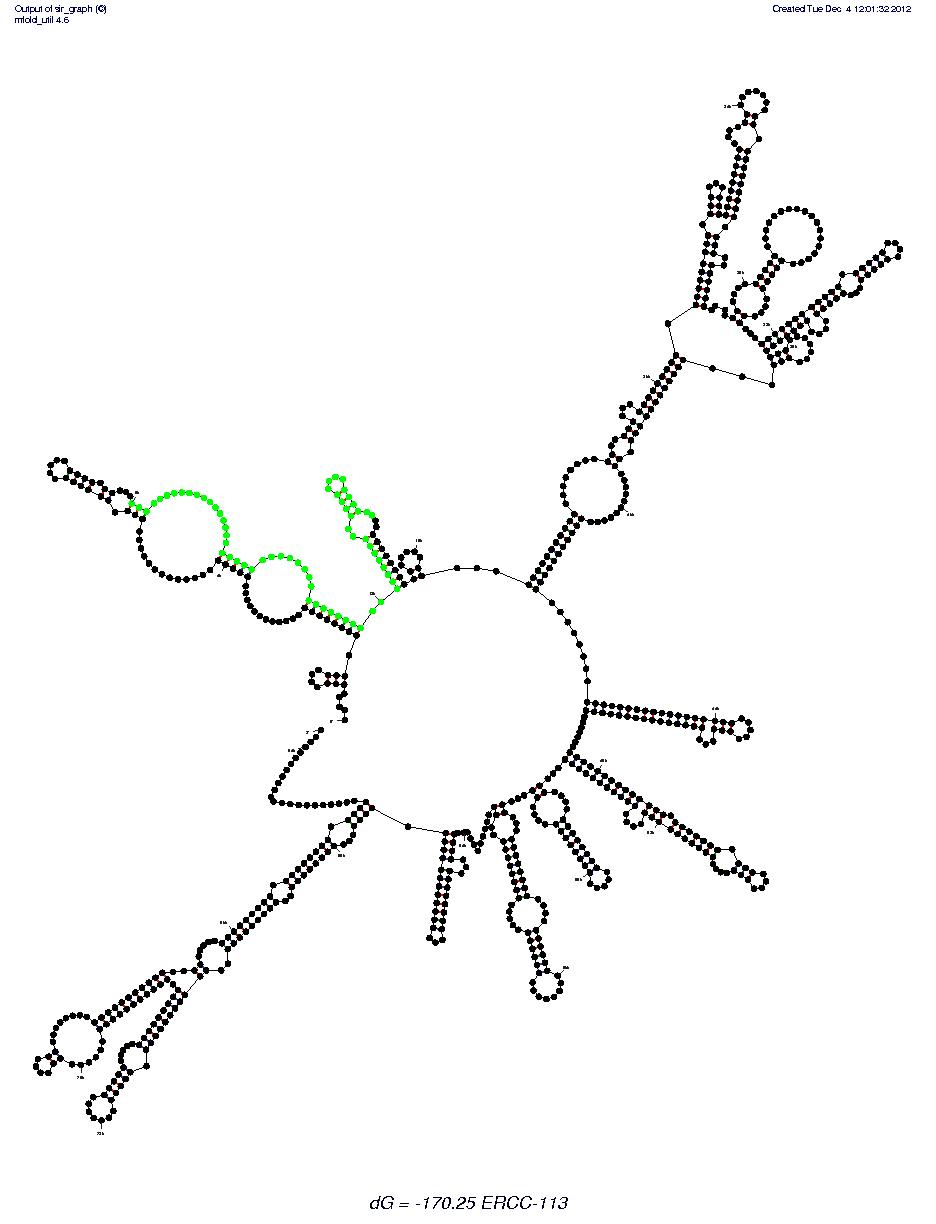


**G**


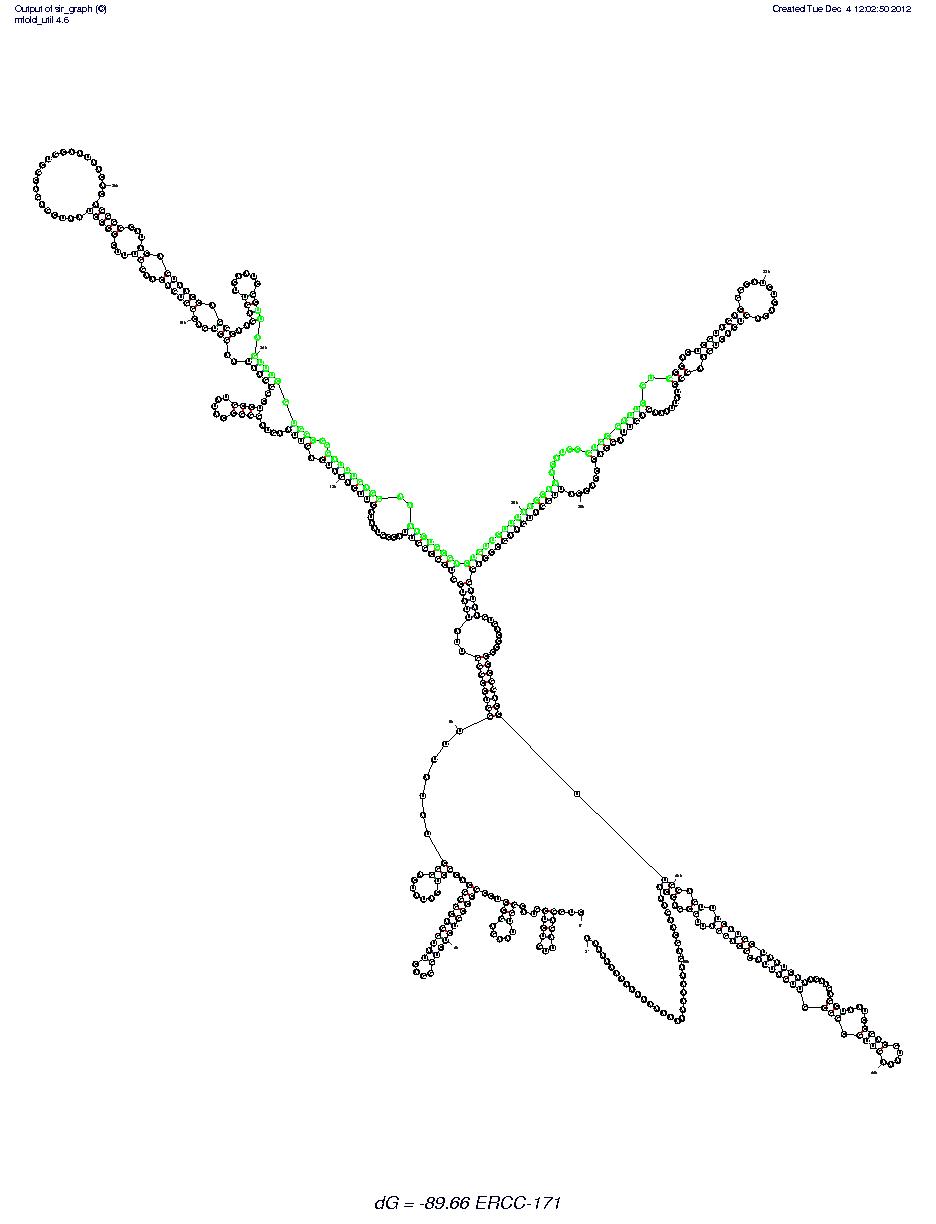


**H**

Supplement: Figure S1 — RNA Secondary Structure Predictions from mFold. (A) MMP1, (B) UBC, (C) ERCC-13, (D) ERCC-25, (E) ERCC-42, (F) ERCC-99, (G) ERCC-113 and (H) ERCC-171. Green highlighted regions indicate amplicon. Folding predictions were performed at 45°C (temperature of RT step). (DOCX) [file pone.0075296.s001.docx]

**Figure S2**


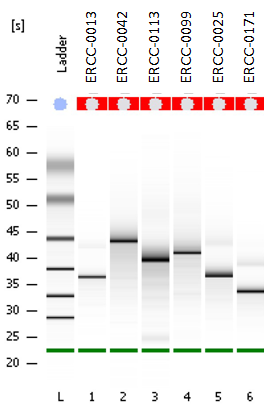

Supplement: Figure S2 — Integrity assessment of Synthetic RNA Transcripts. 2100 Bioanalyzer quantification for all six synthetic targets was comparable to nanodrop concentration estimates (p = 0.660, with an average fold change between the two measurements of 1.02). (DOCX) [file pone.0075296.s002.docx]
