# Supplementary material for: Evaluation of Digital PCR for Absolute RNA Quantification
Source: PLoS One. 2013 Sep 20;8(9):e75296. doi: 10.1371/journal.pone.0075296 (PMC3779174; doi:10.1371/journal.pone.0075296)

**Figure S3**

**A**

Ambion ERCC-25 Ambion ERCC-99 Ambion ERCC-25 & -99 (duplex) Ambion ERCC-25 & -99 (NTC)


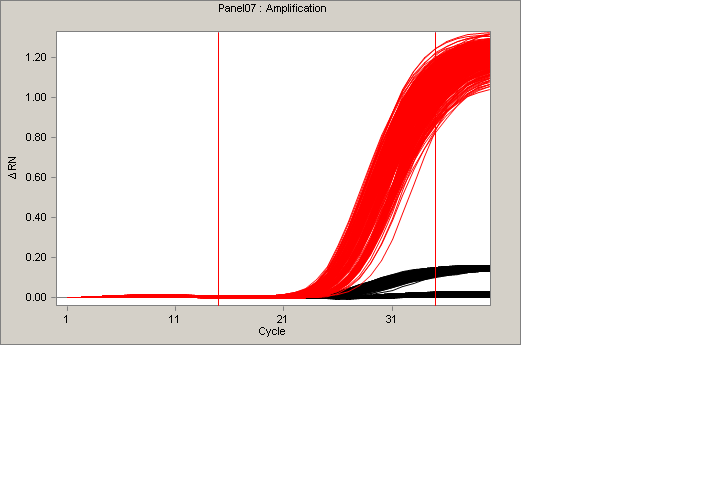

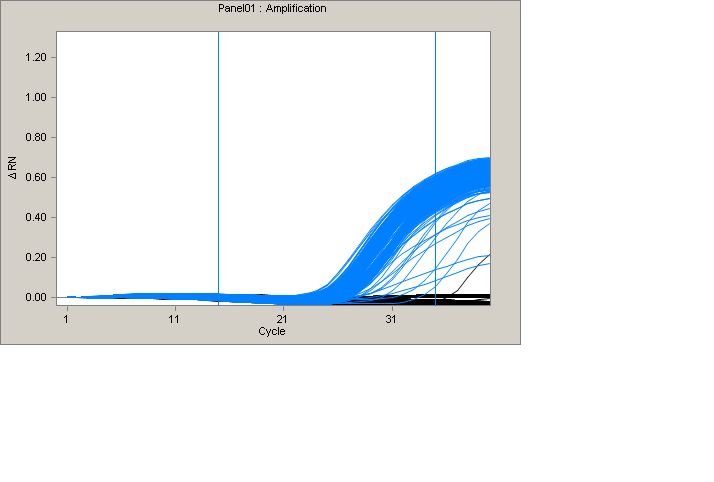

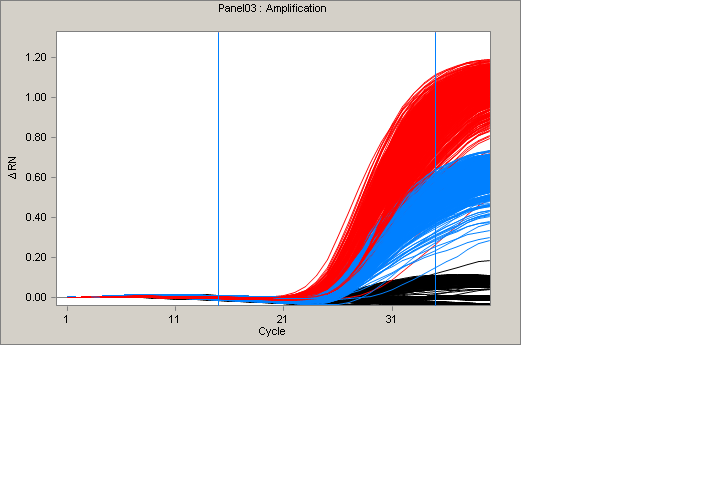

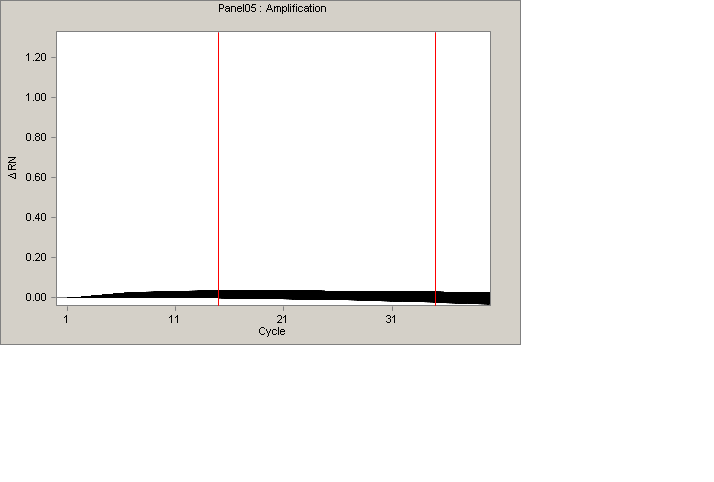


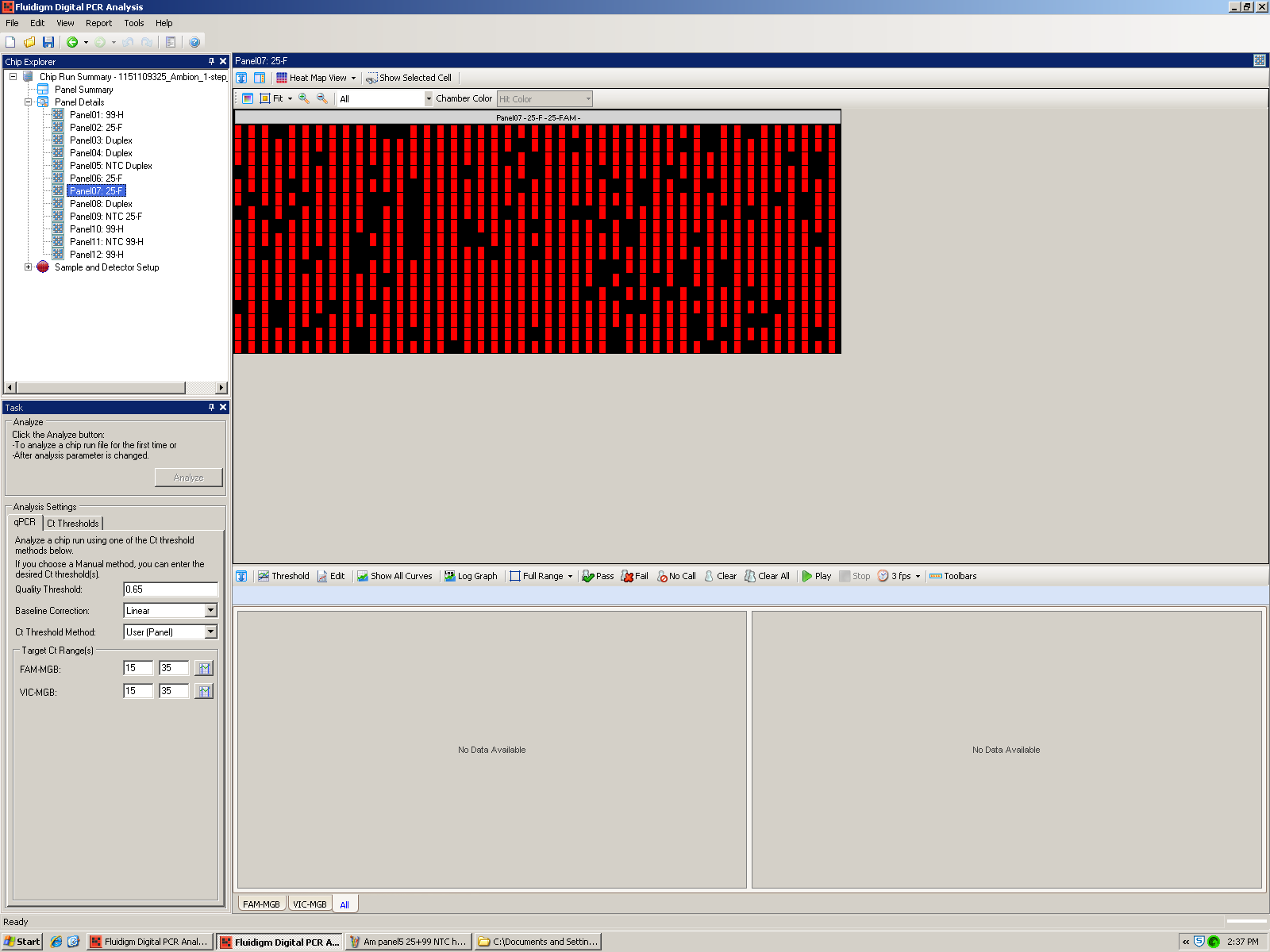

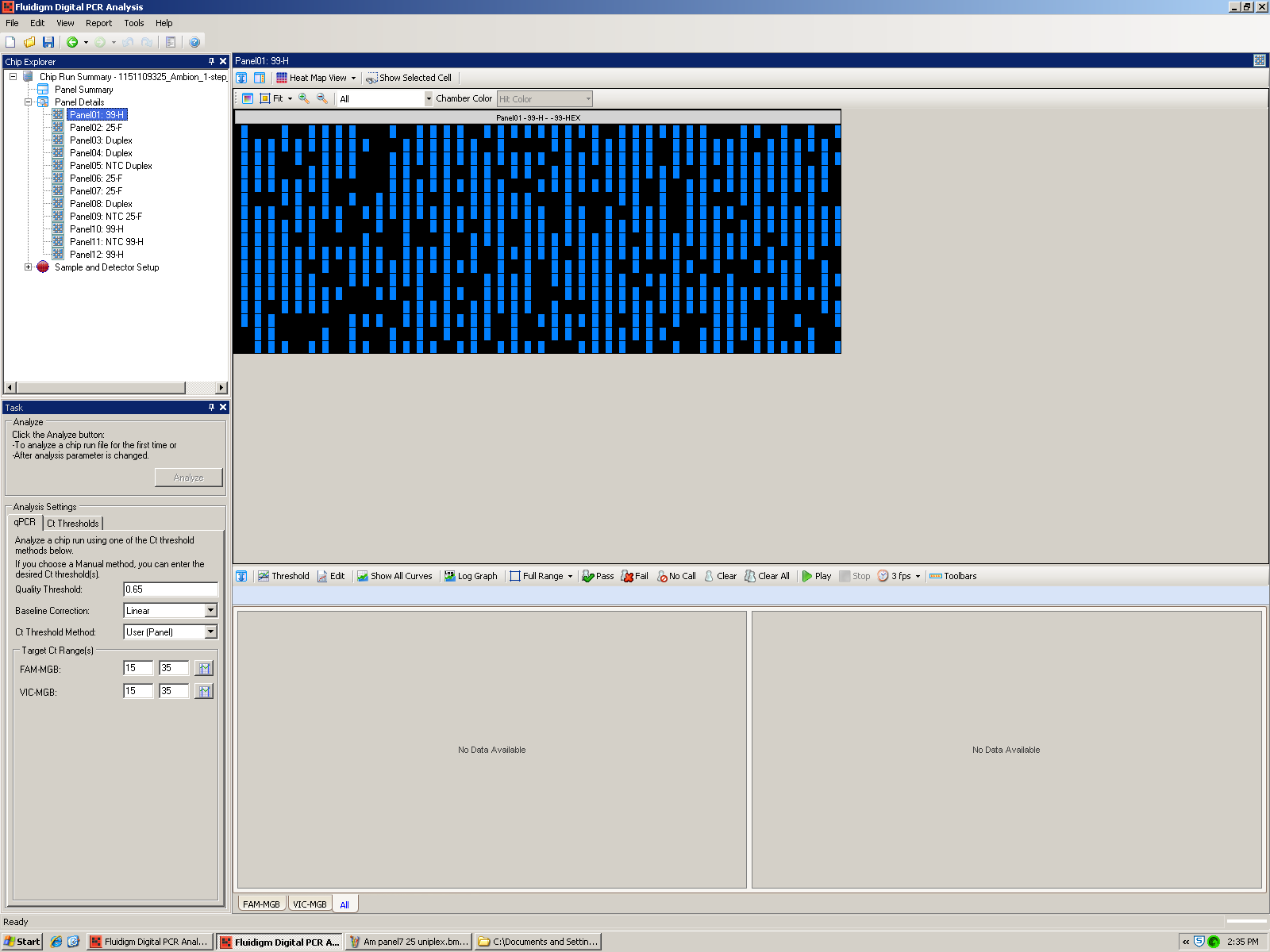

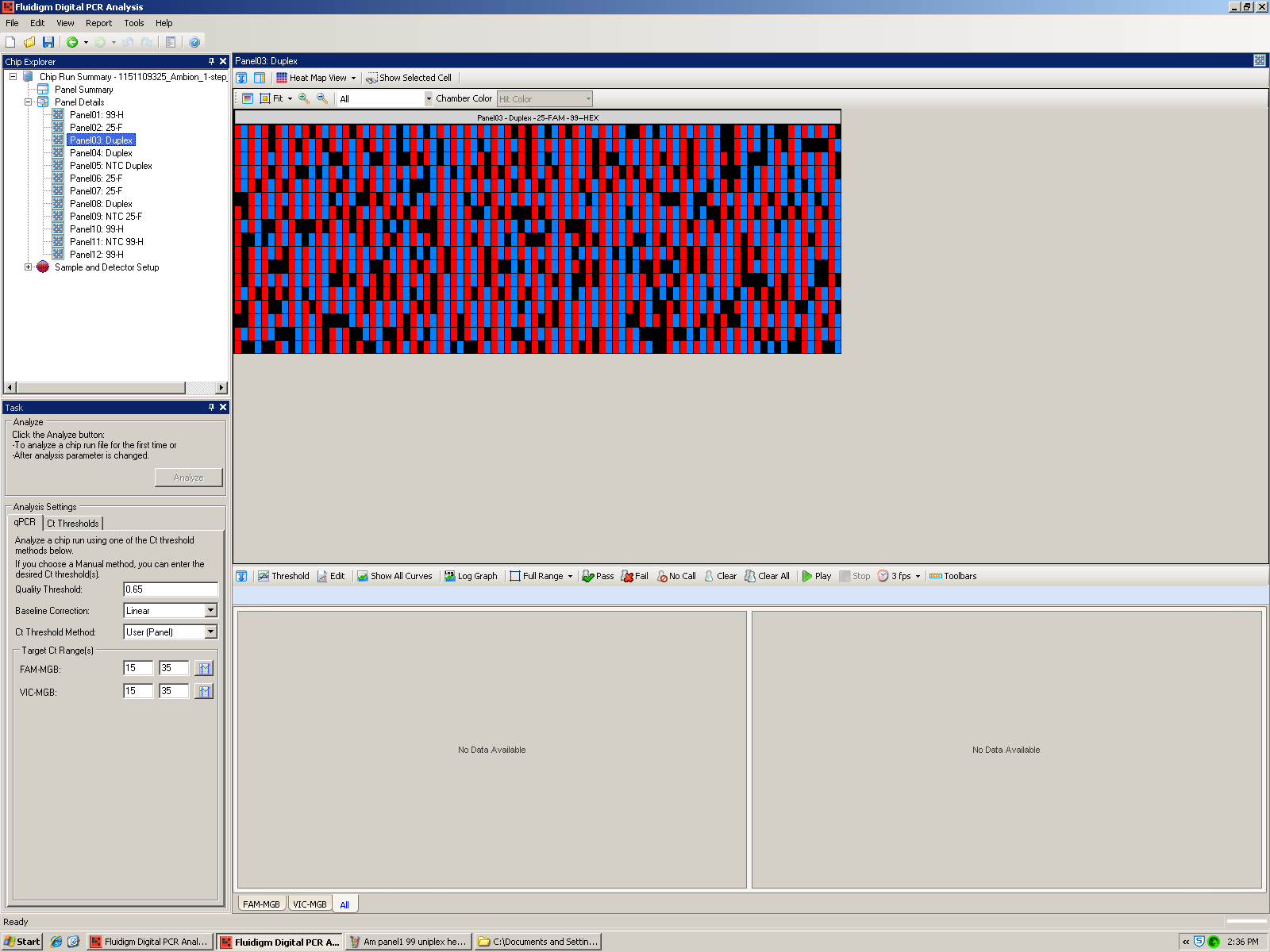

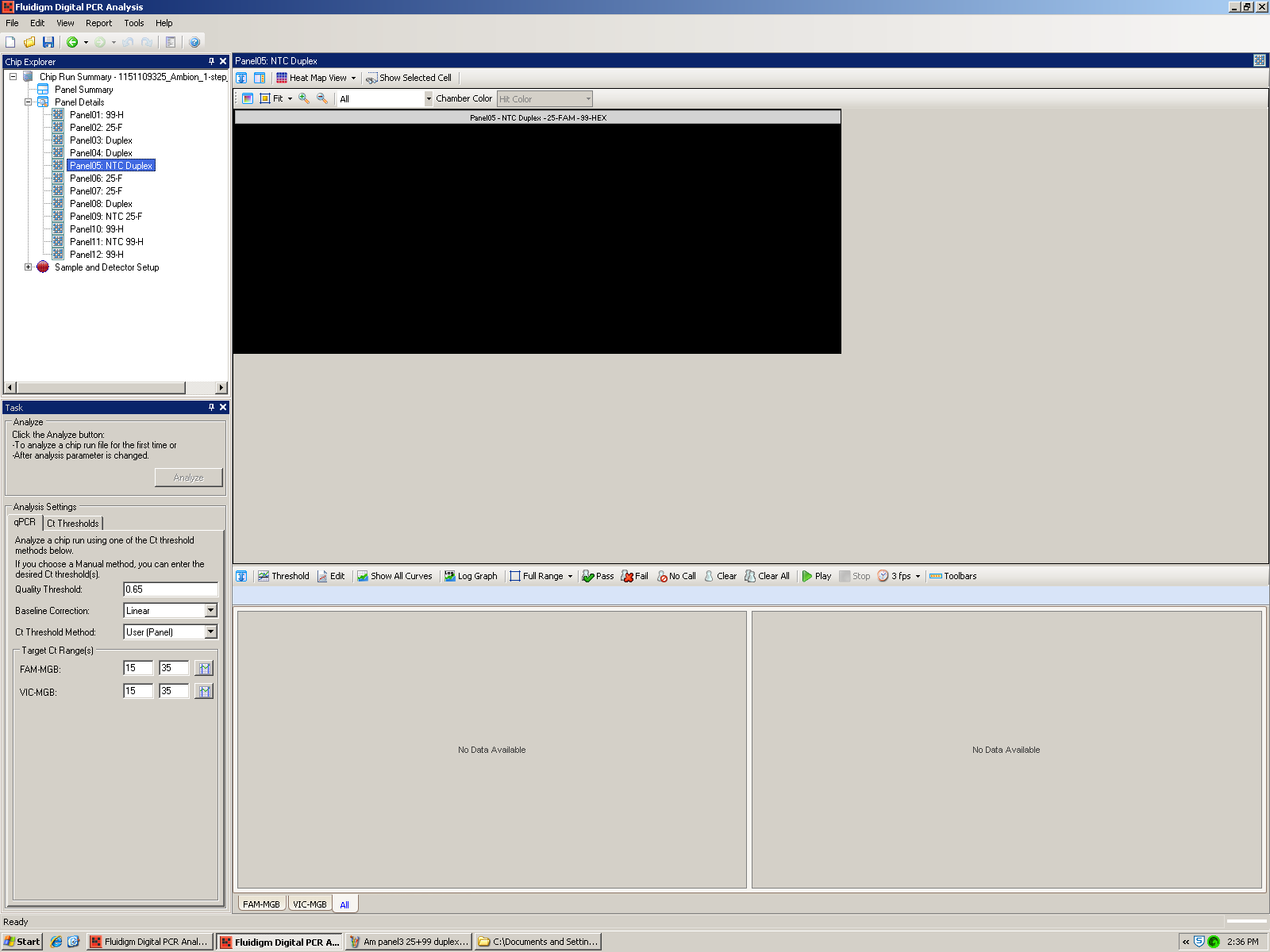


Invitrogen ERCC-25 Invitrogen ERCC-99 Invitrogen ERCC-25 & -99 (duplex) Invitrogen ERCC-25 & -99 (NTC)


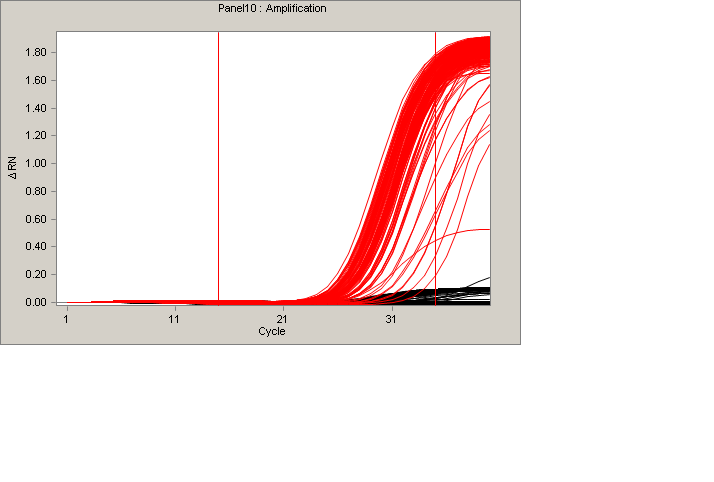

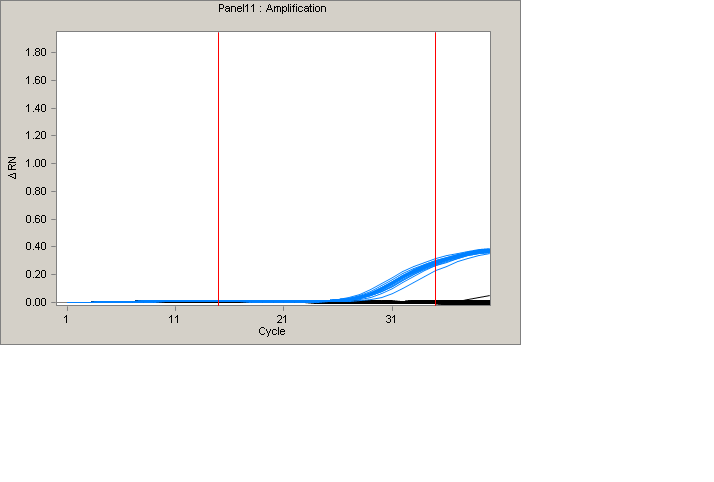

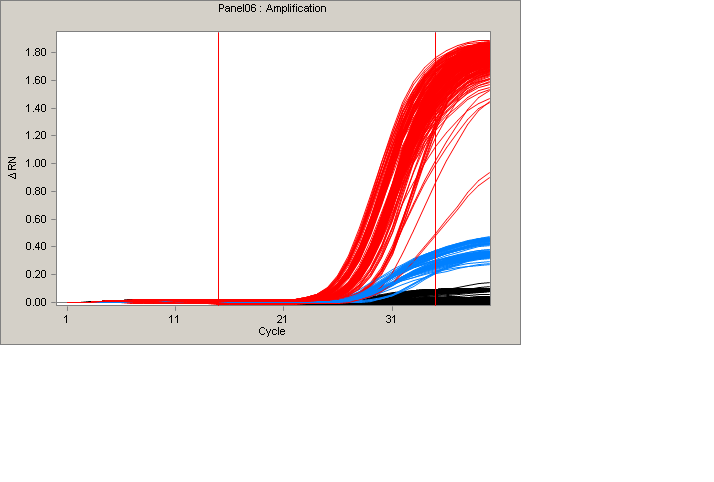

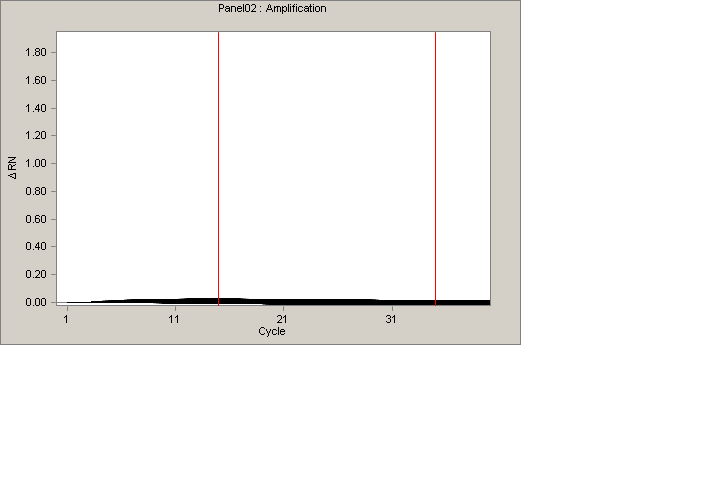


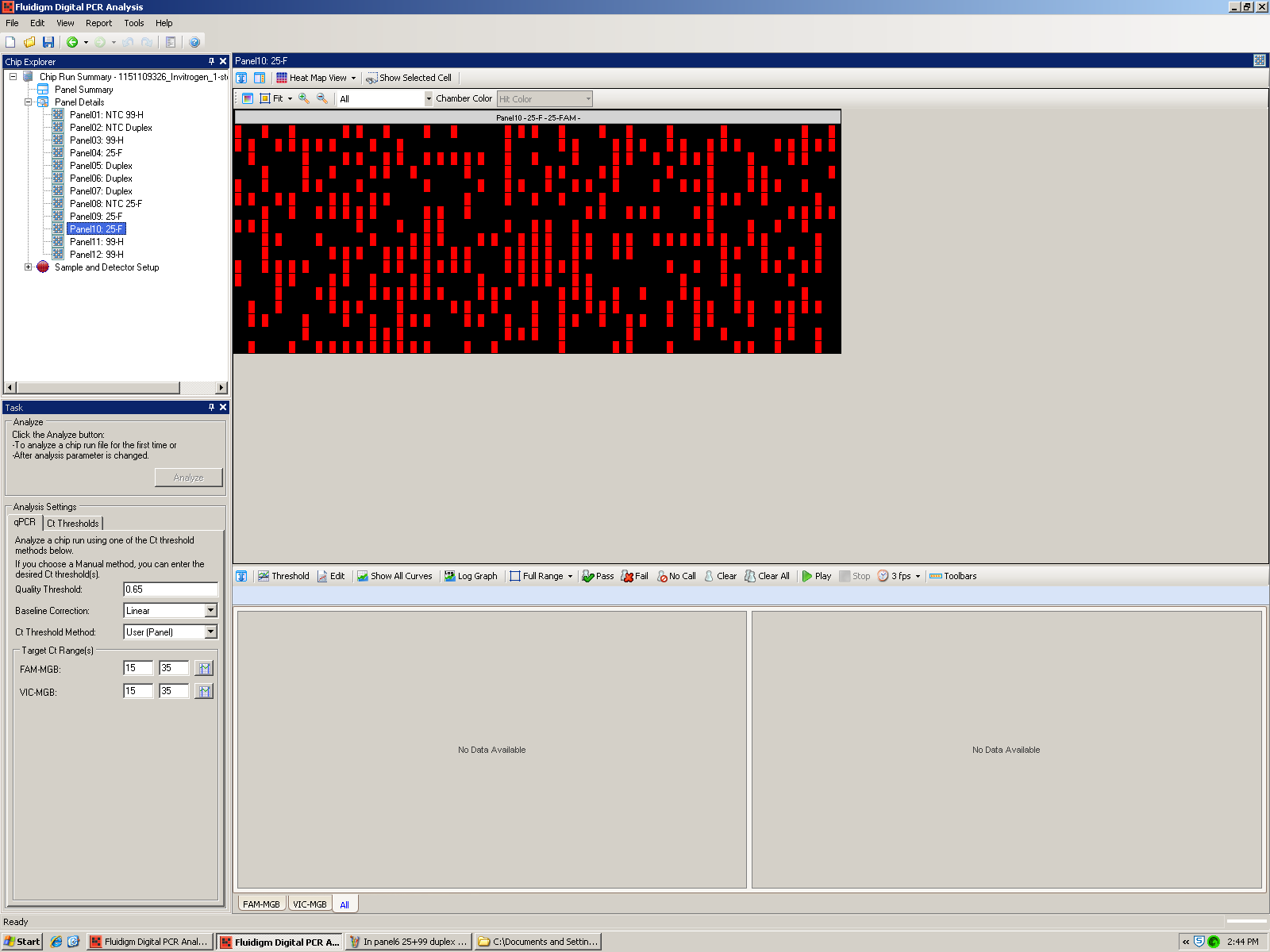

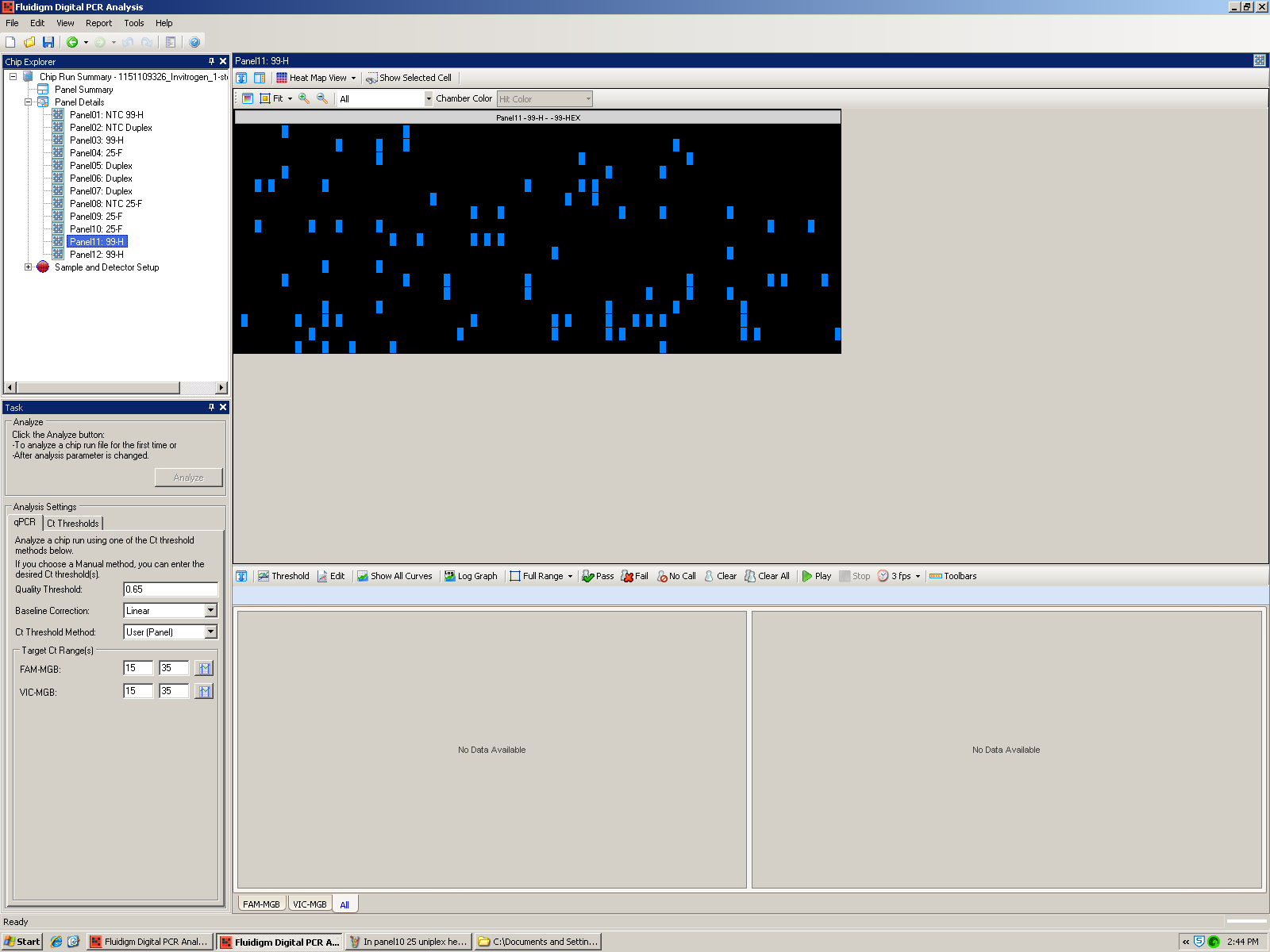

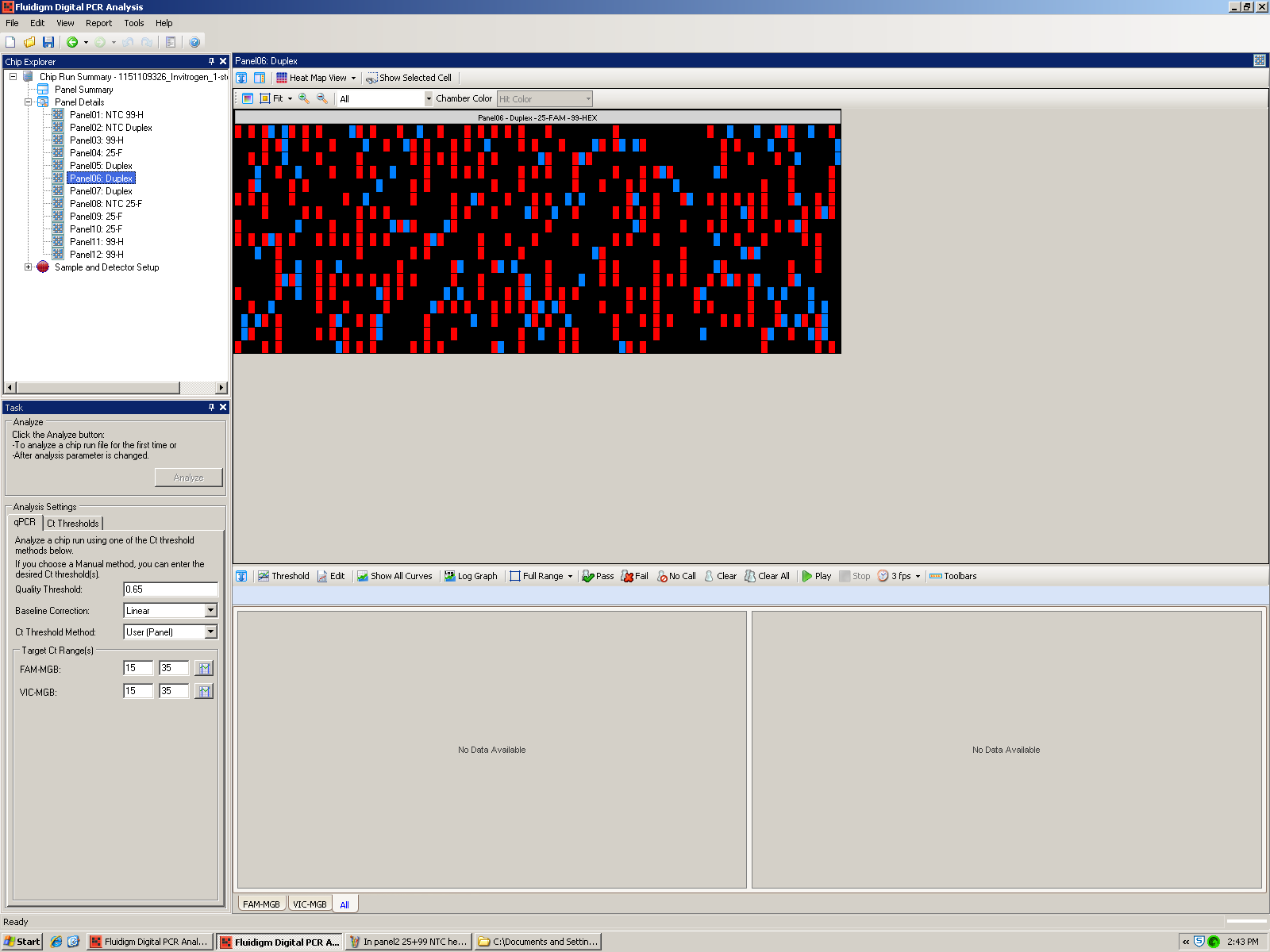

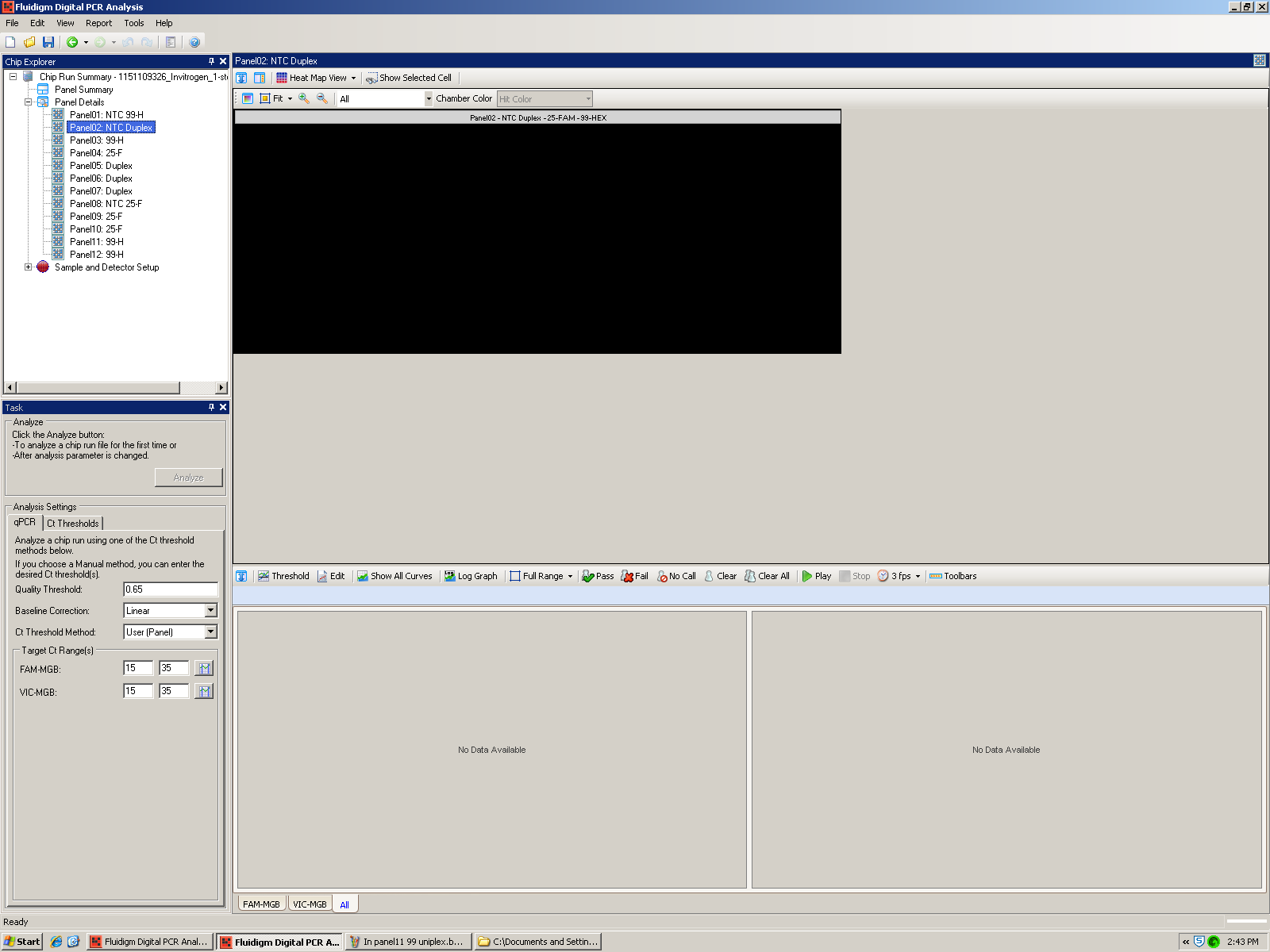


Qiagen ERCC-25 Qiagen ERCC-99 Qiagen ERCC-25 & -99 (duplex) Qiagen ERCC-25 & -99 (NTC)


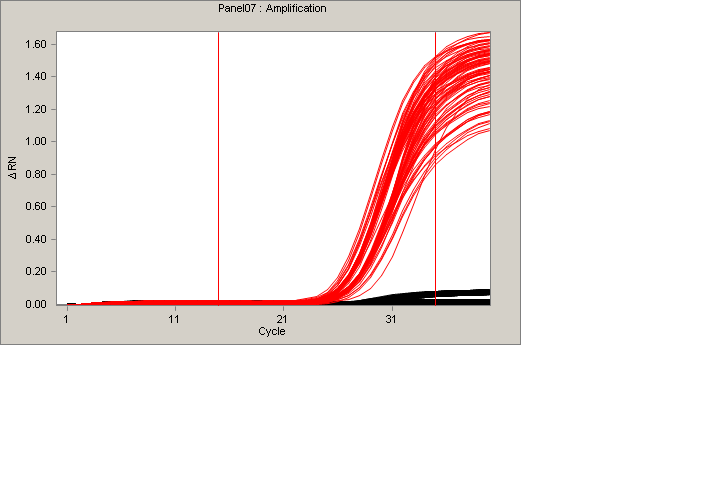

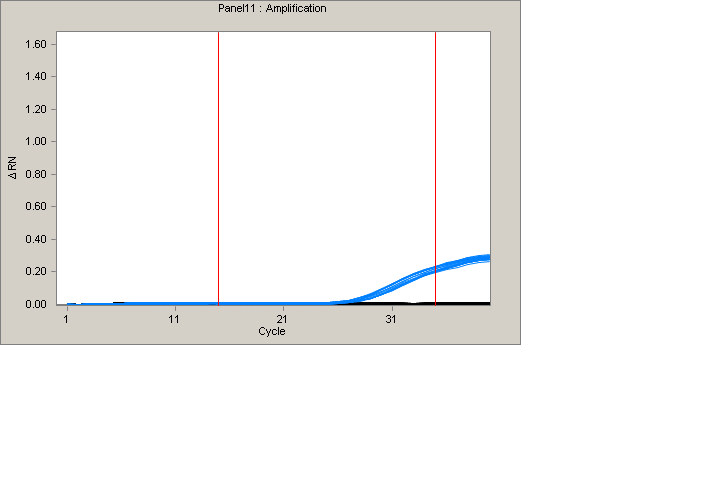

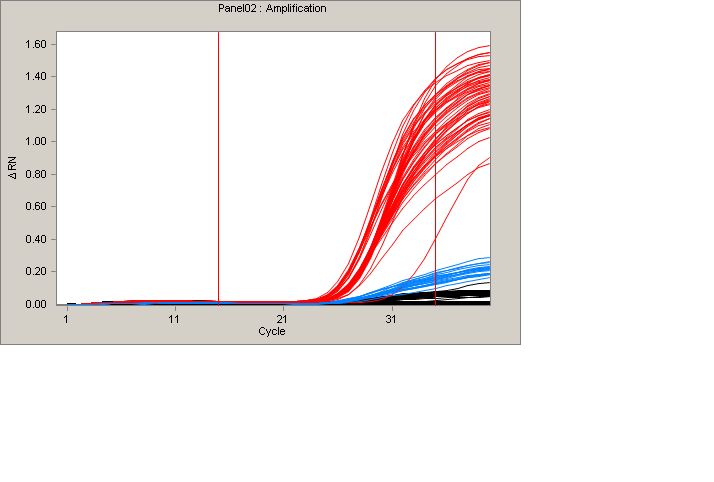

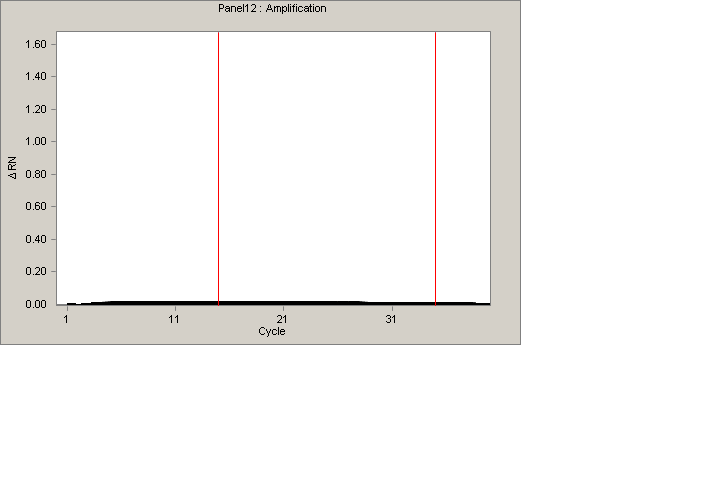


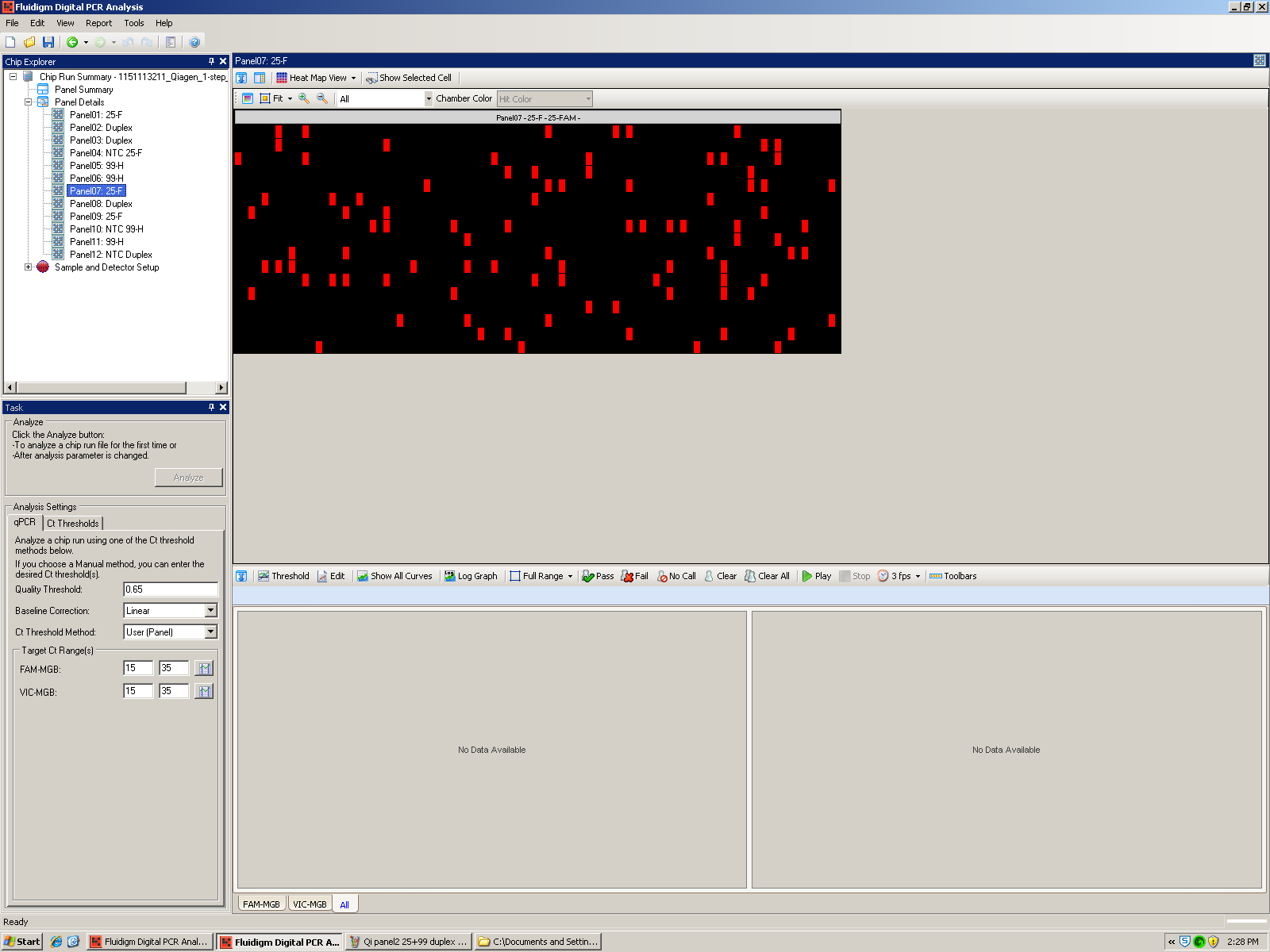

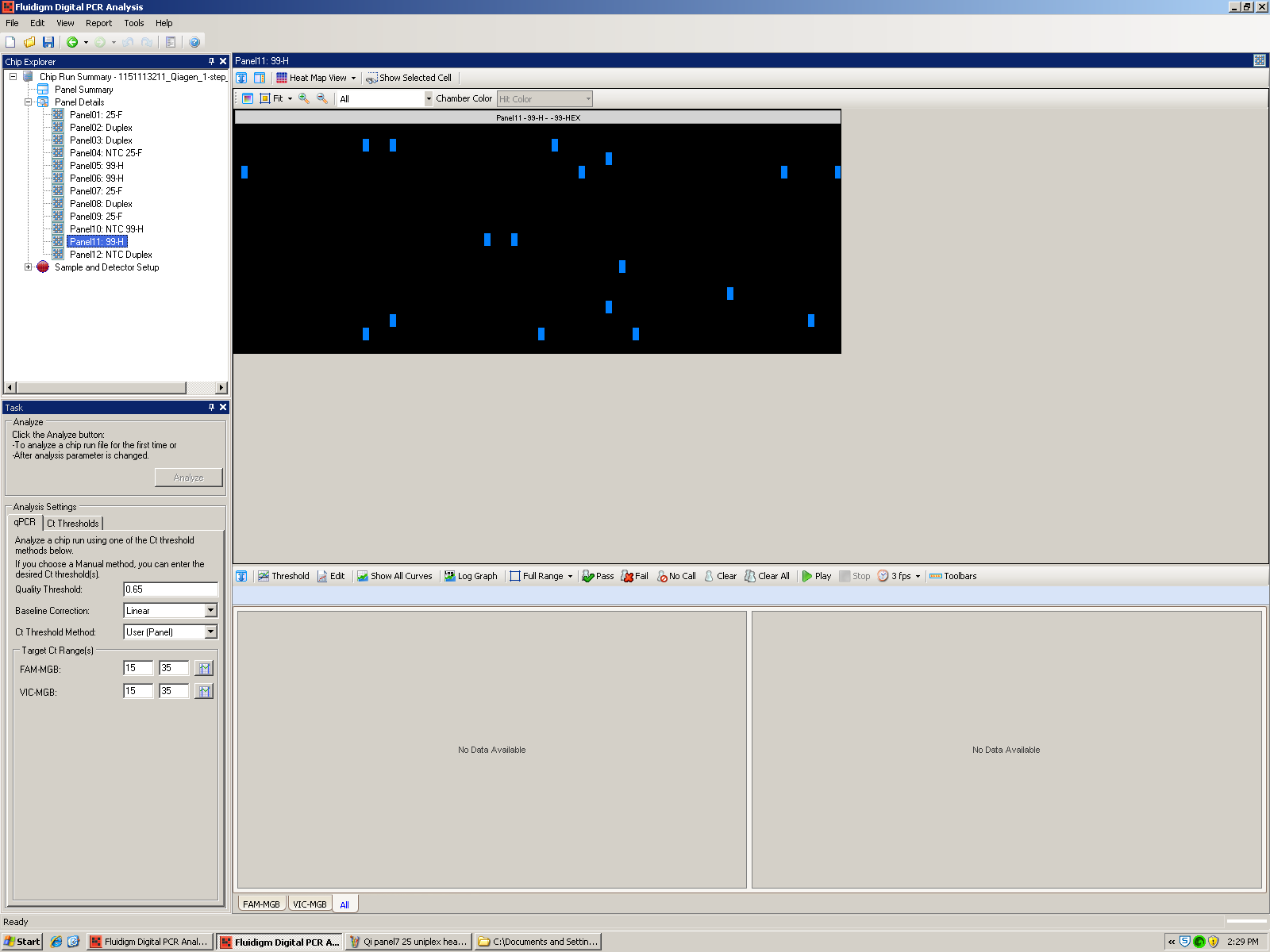

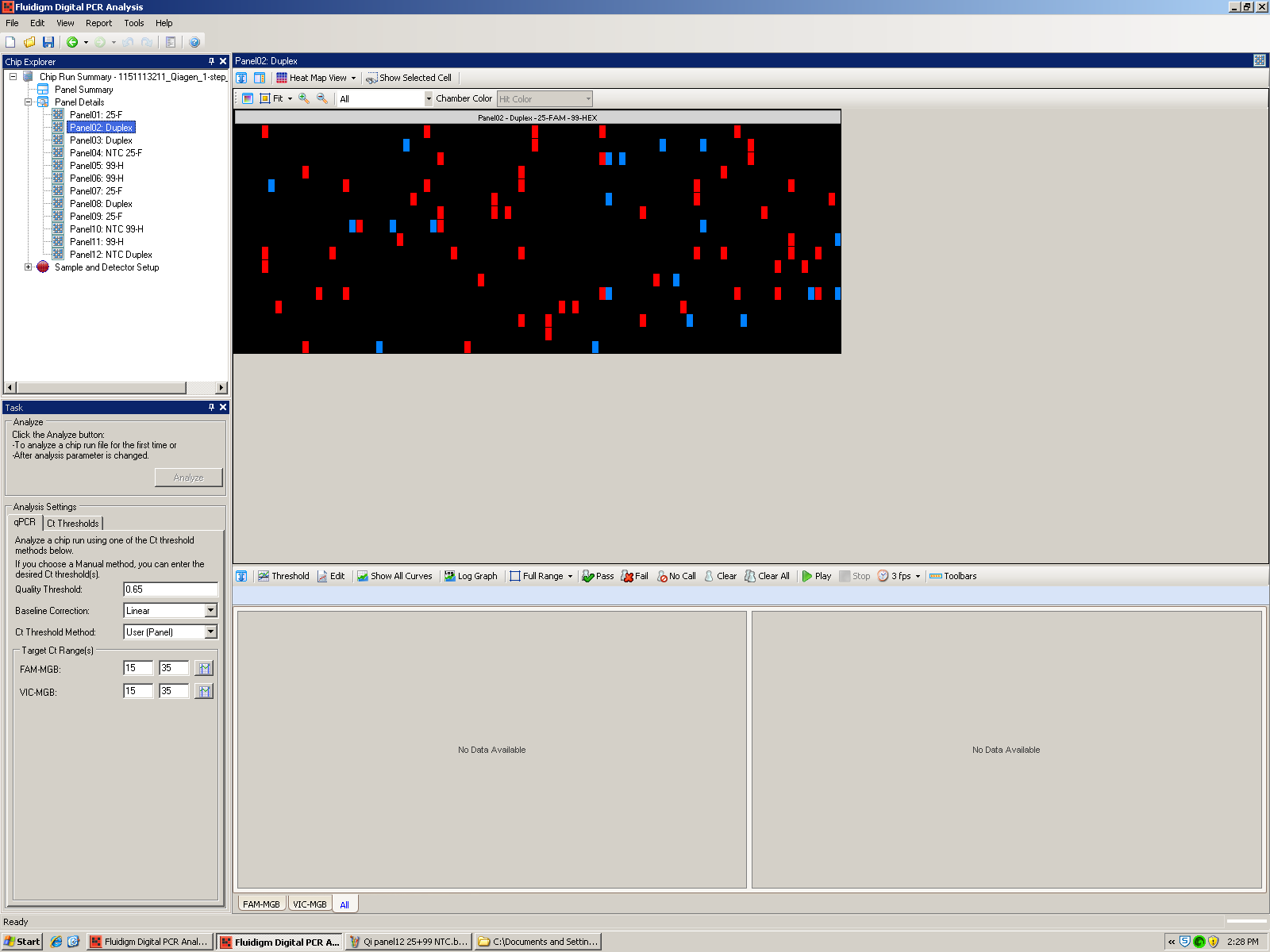

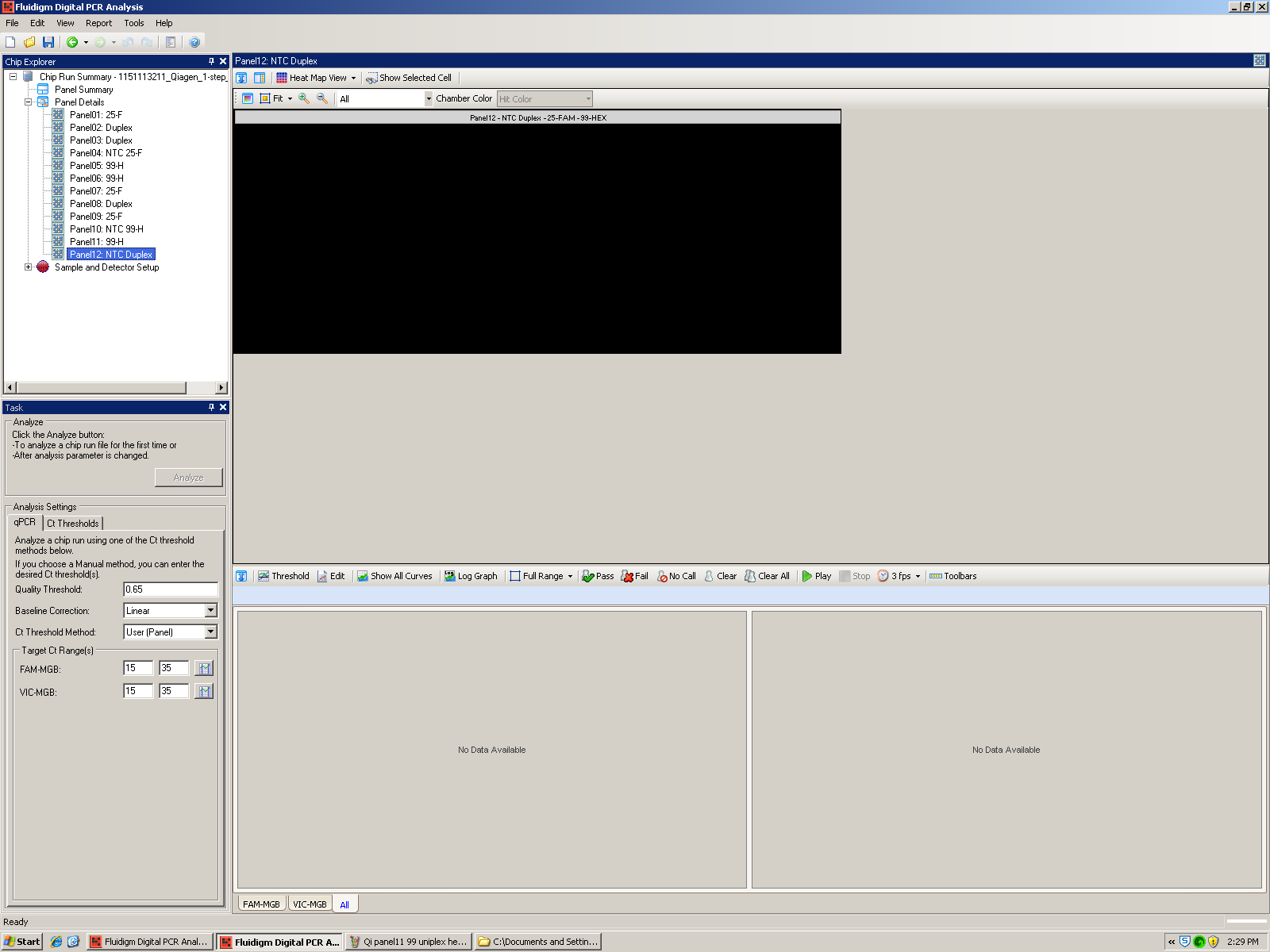


**B**

Ambion ERCC-25 & -99 Ambion MMP1 & UBC Ambion ERCC-25 & UBC Ambion NTC


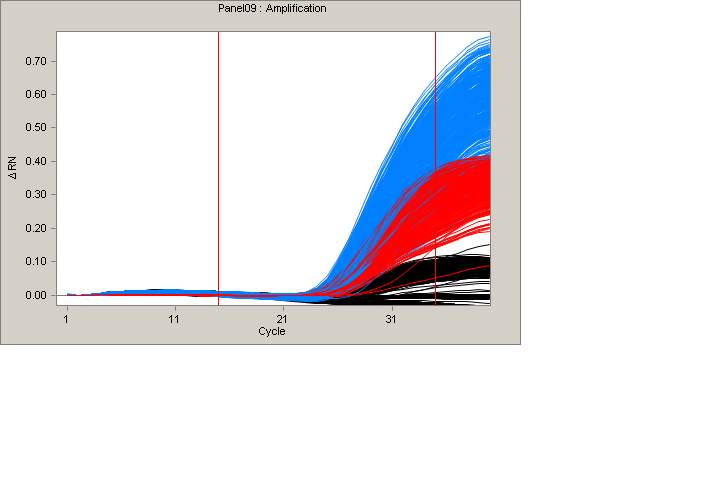

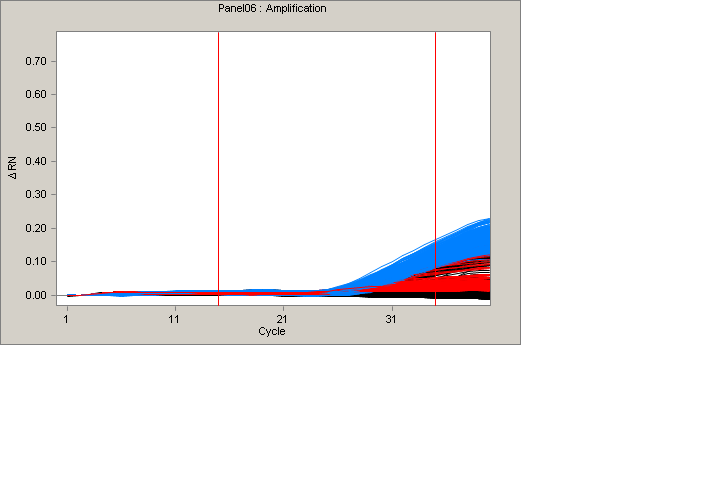

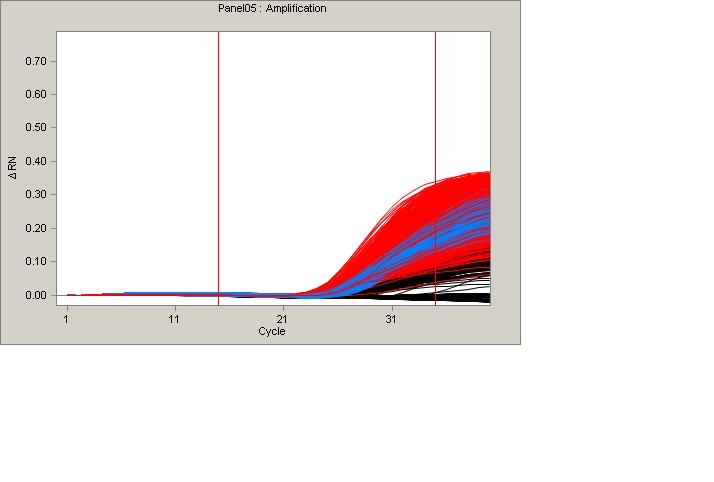

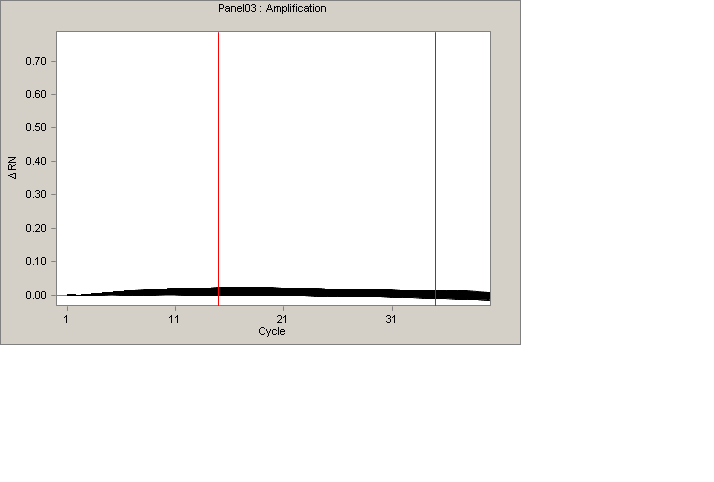


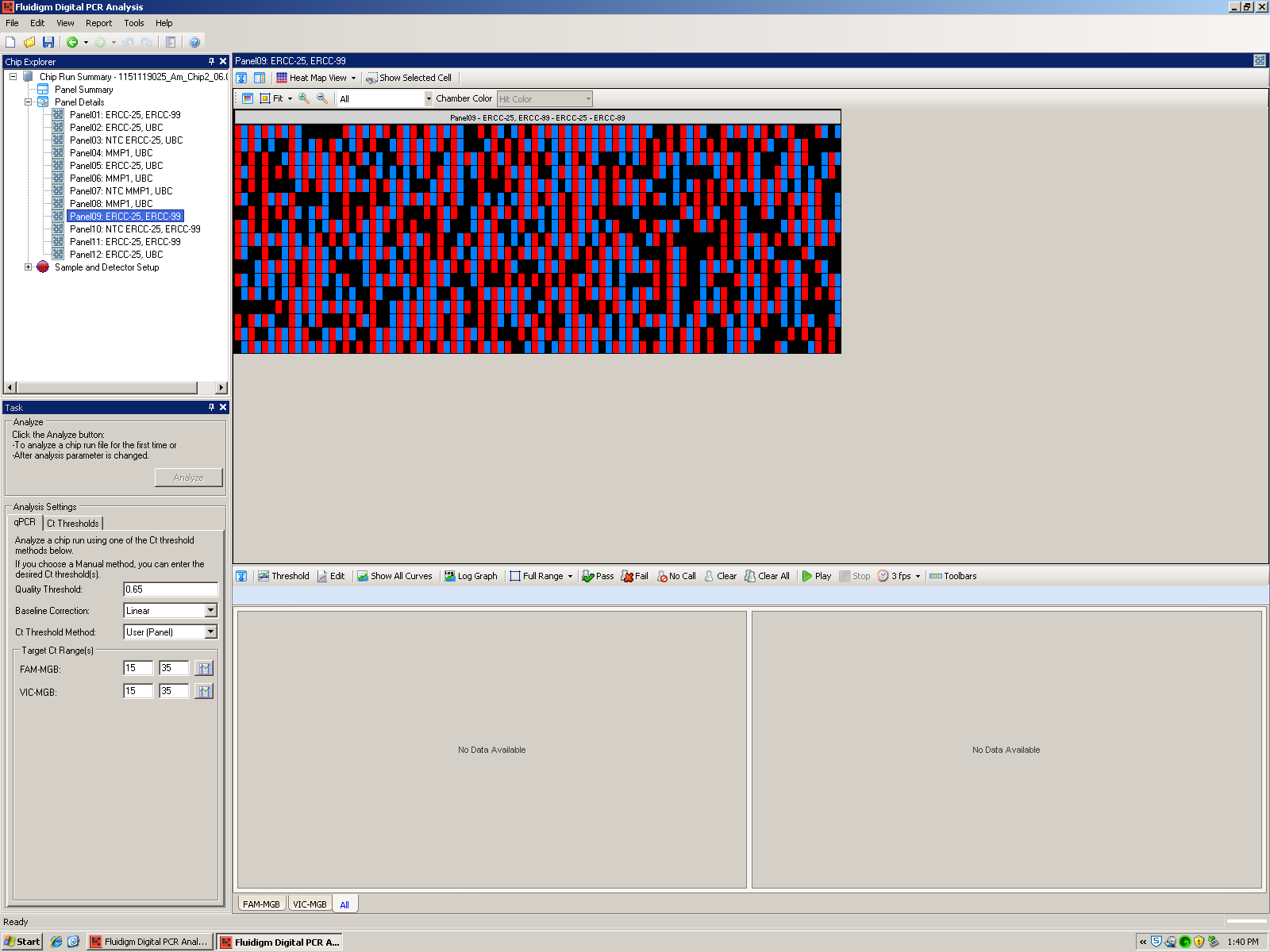

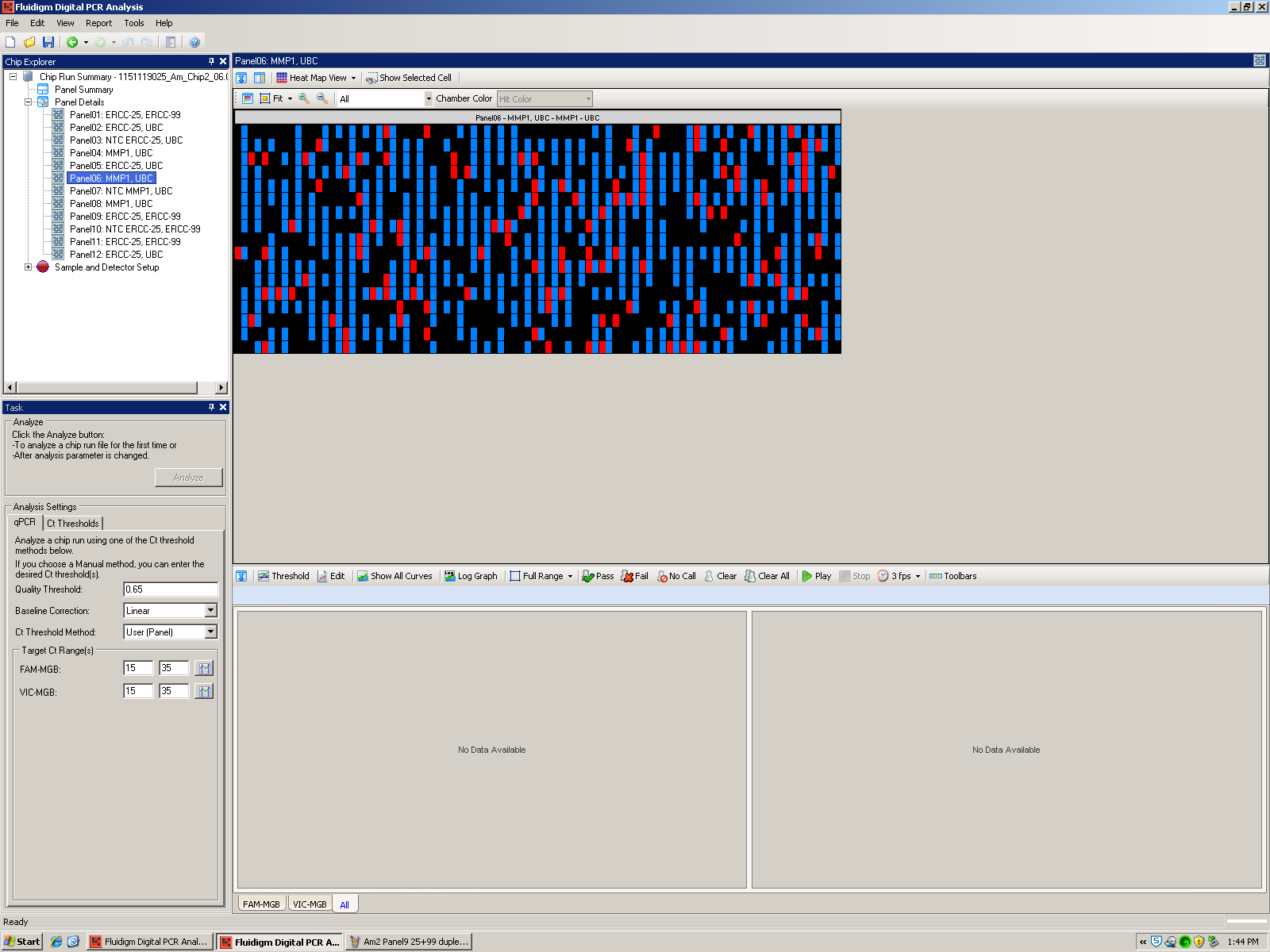

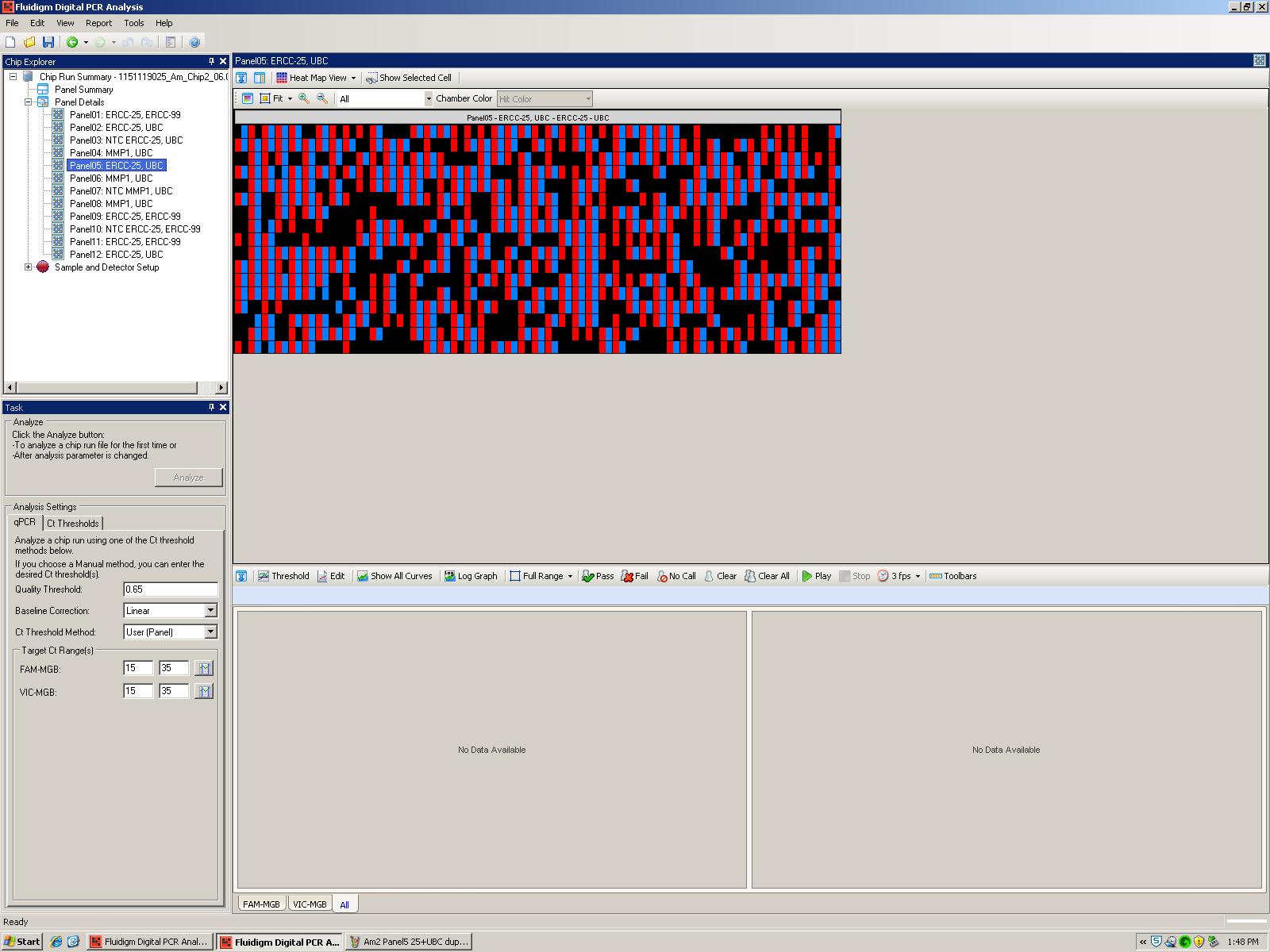

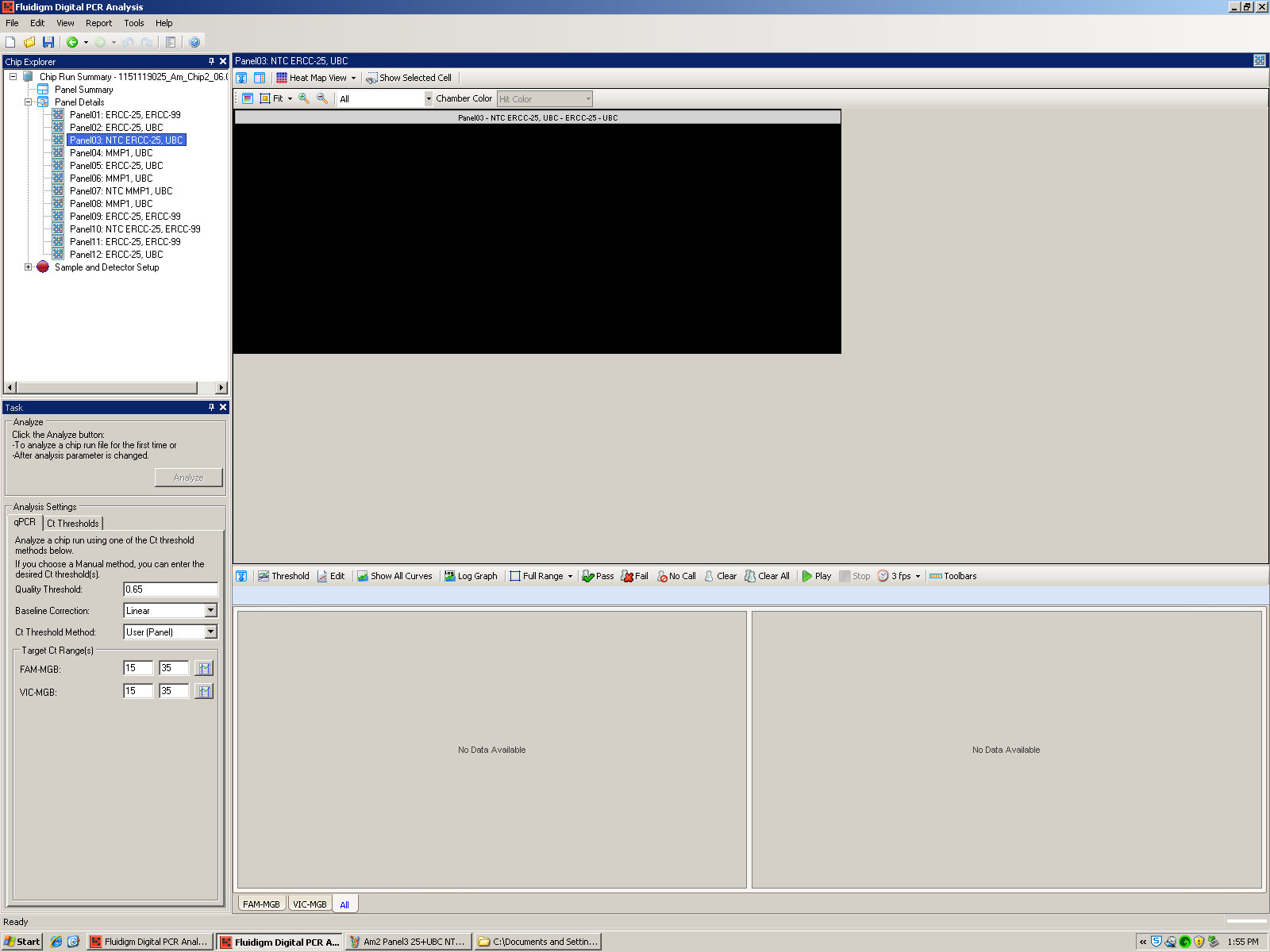


Invitrogen ERCC-25 & -99 Invitrogen MMP1 & UBC Invitrogen ERCC-25 & UBC Invitrogen NTC


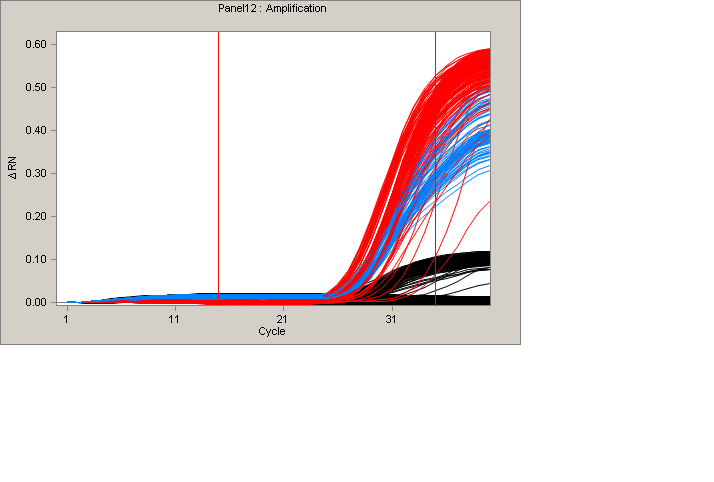

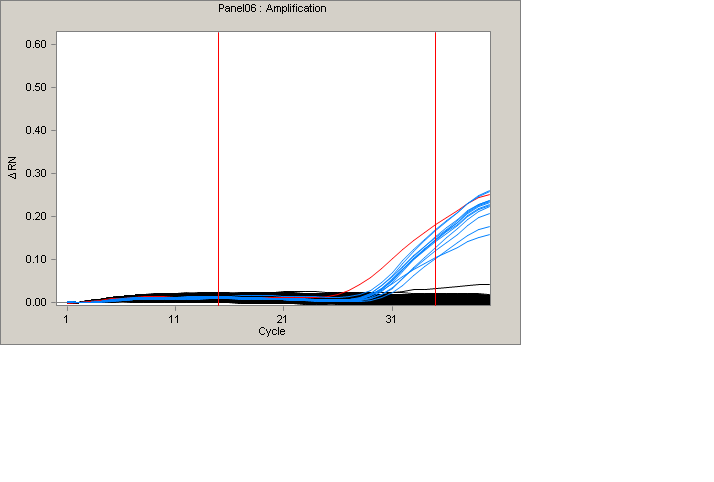

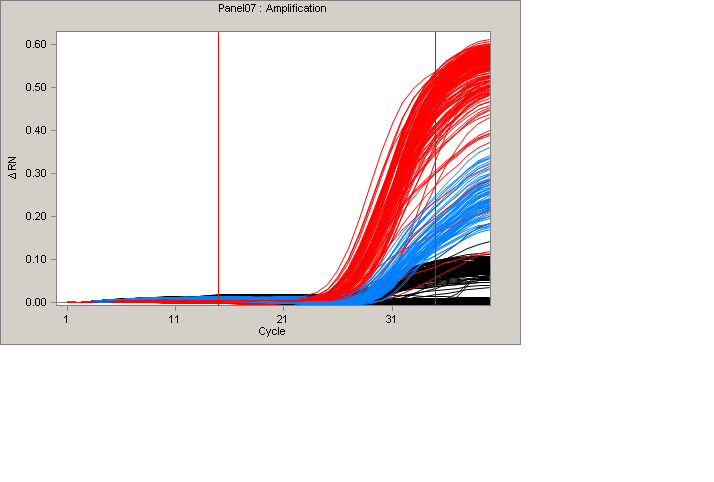

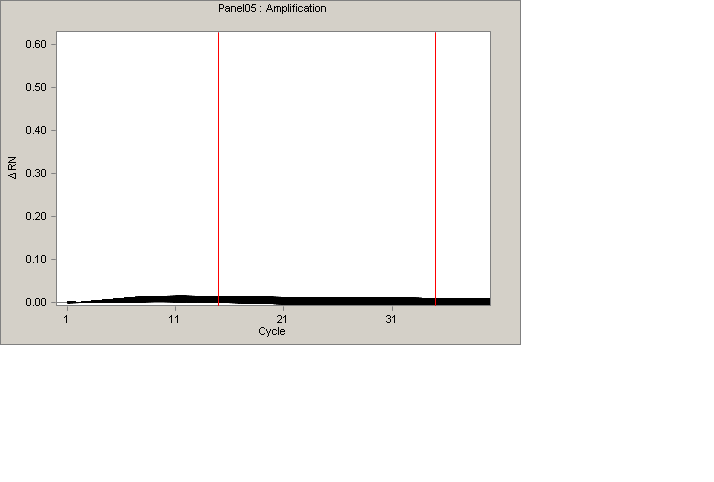


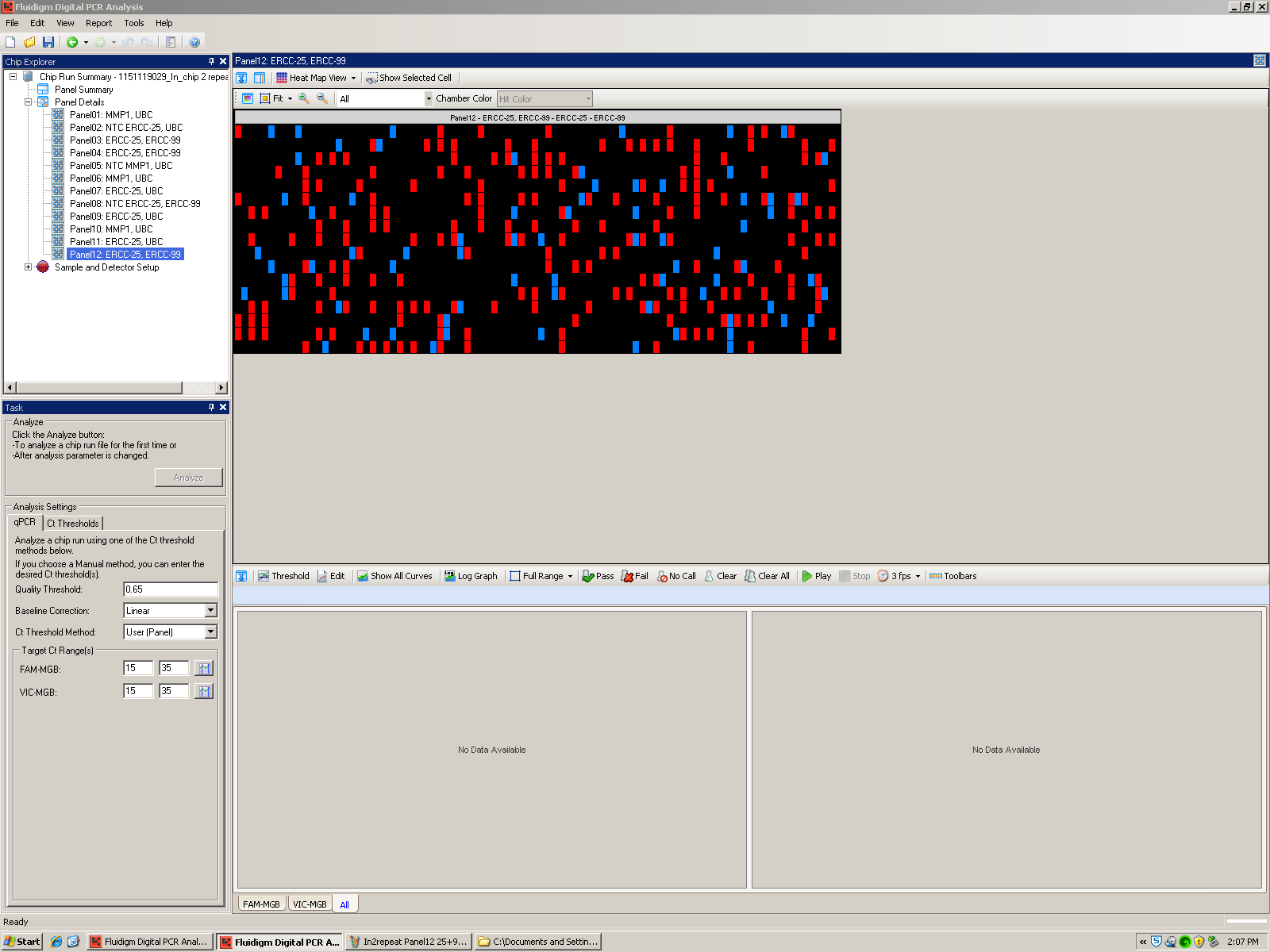

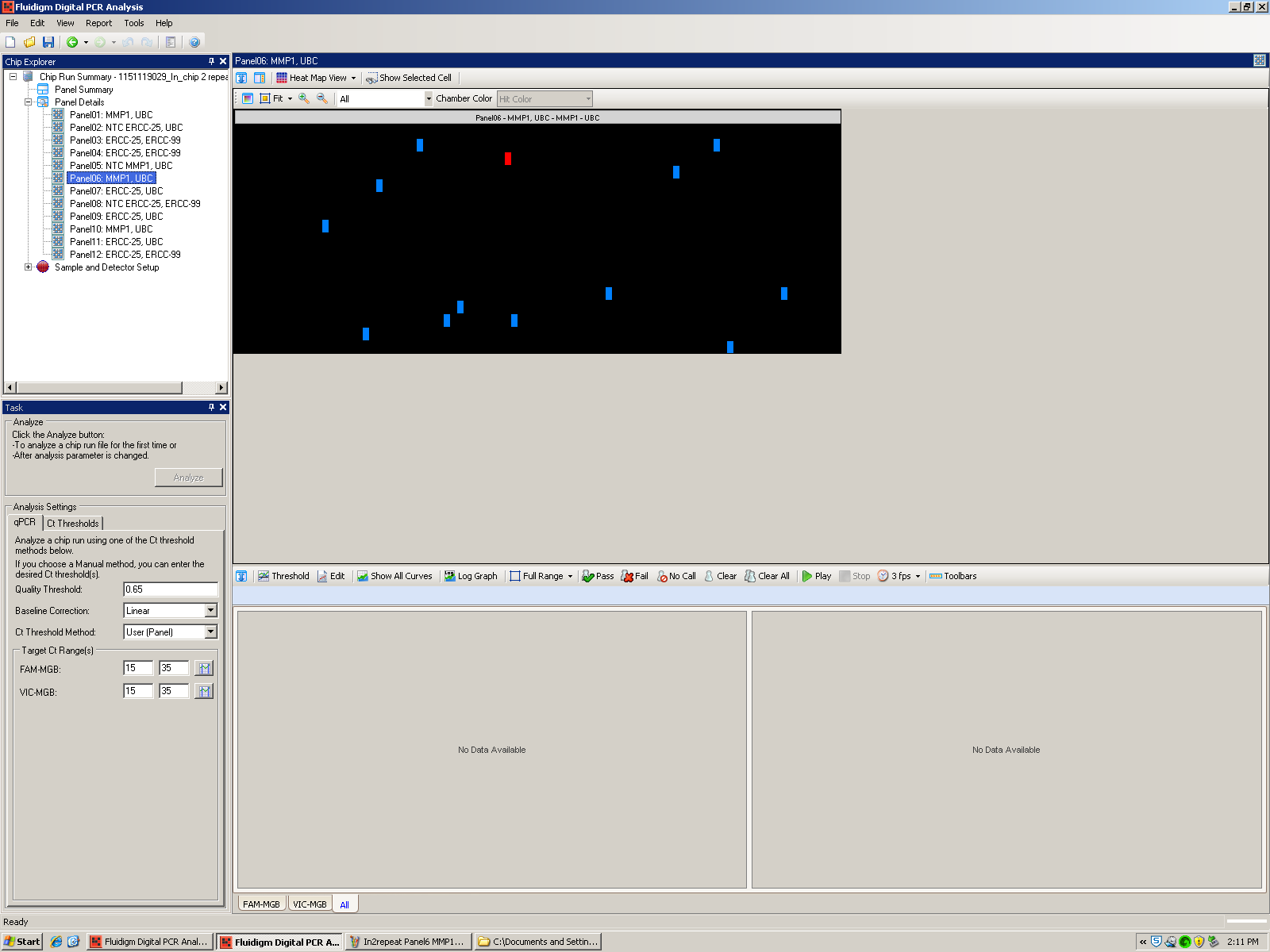

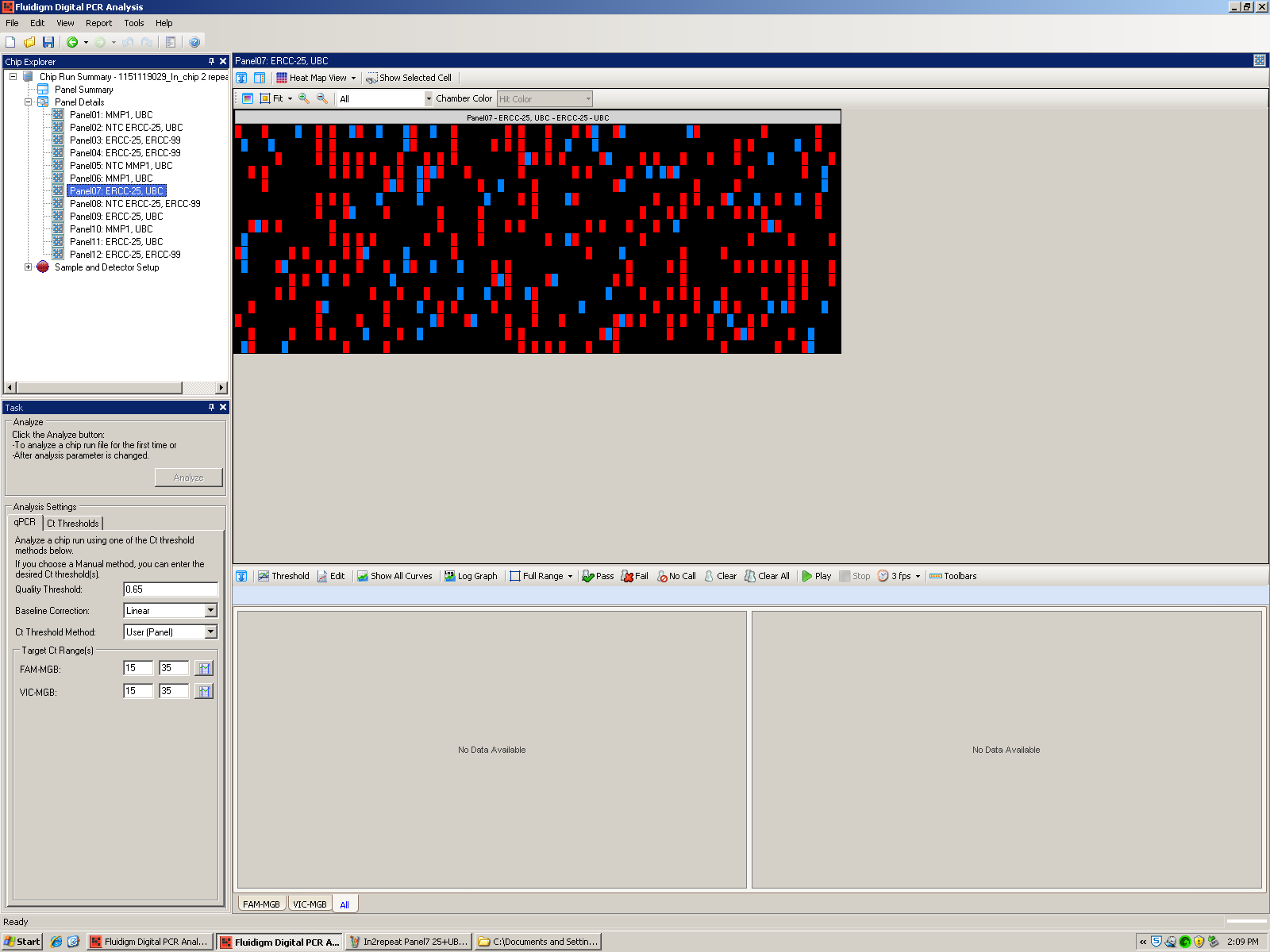

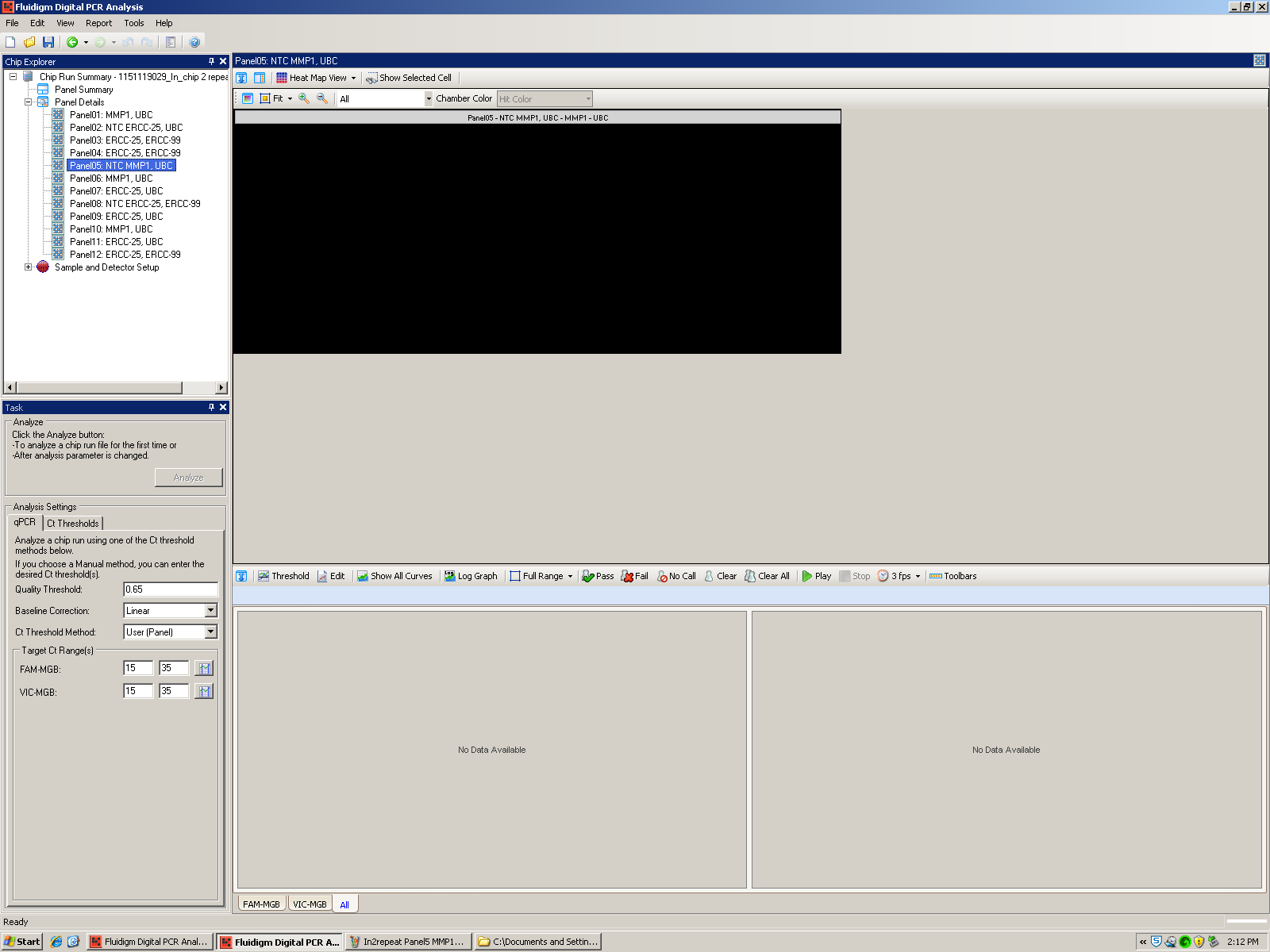


Qiagen ERCC-25 & -99 Qiagen MMP1 & UBC Qiagen ERCC-25 & UBC Qiagen NTC


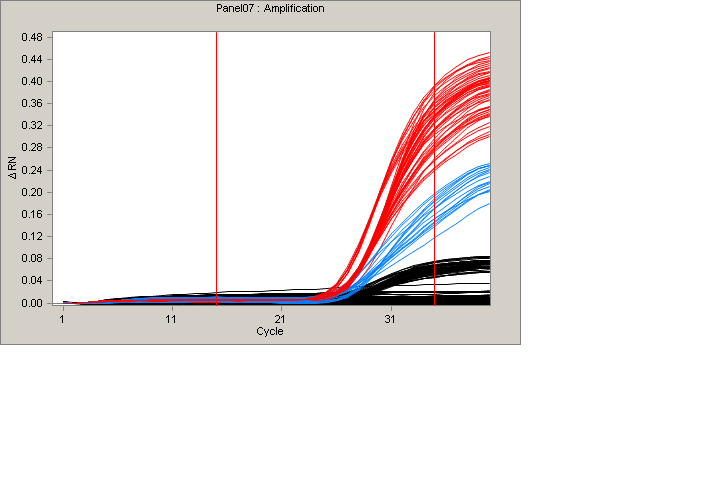

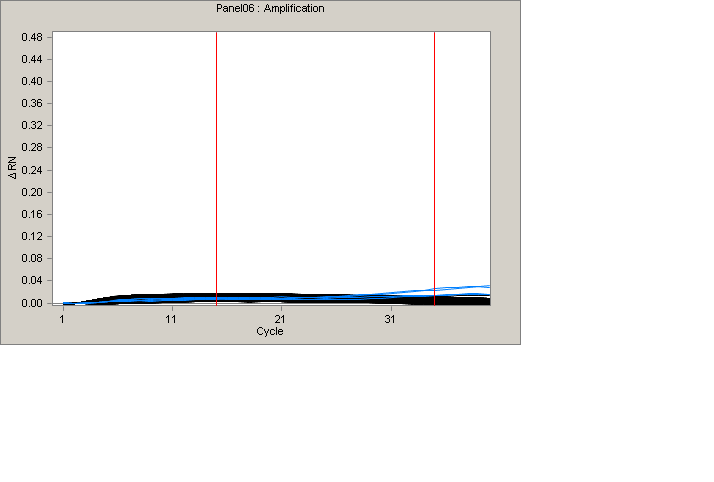

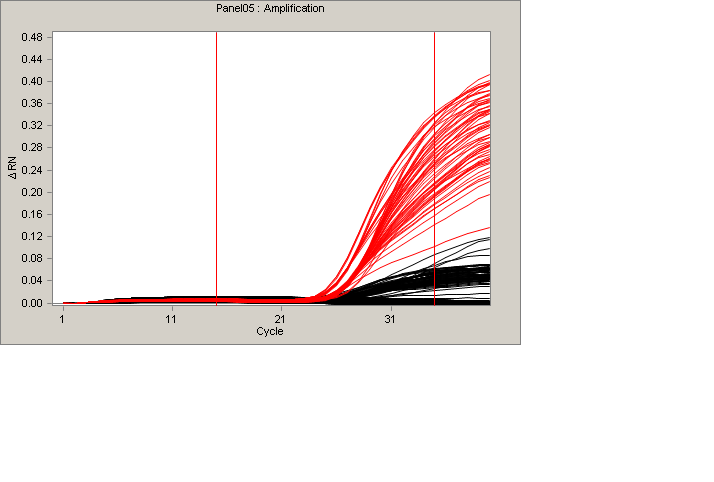

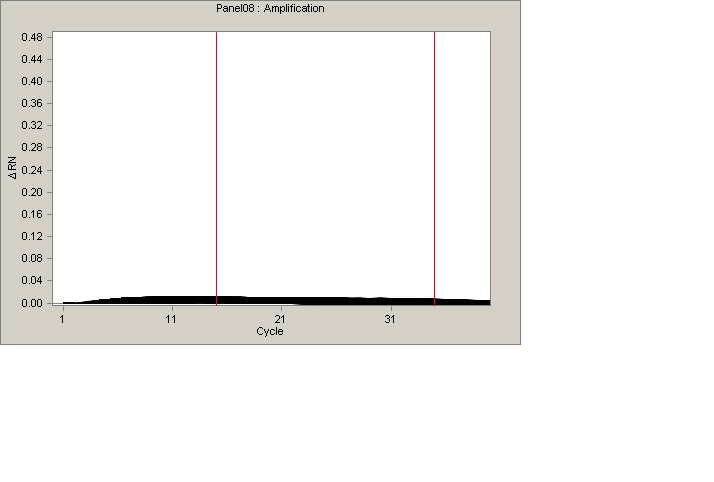


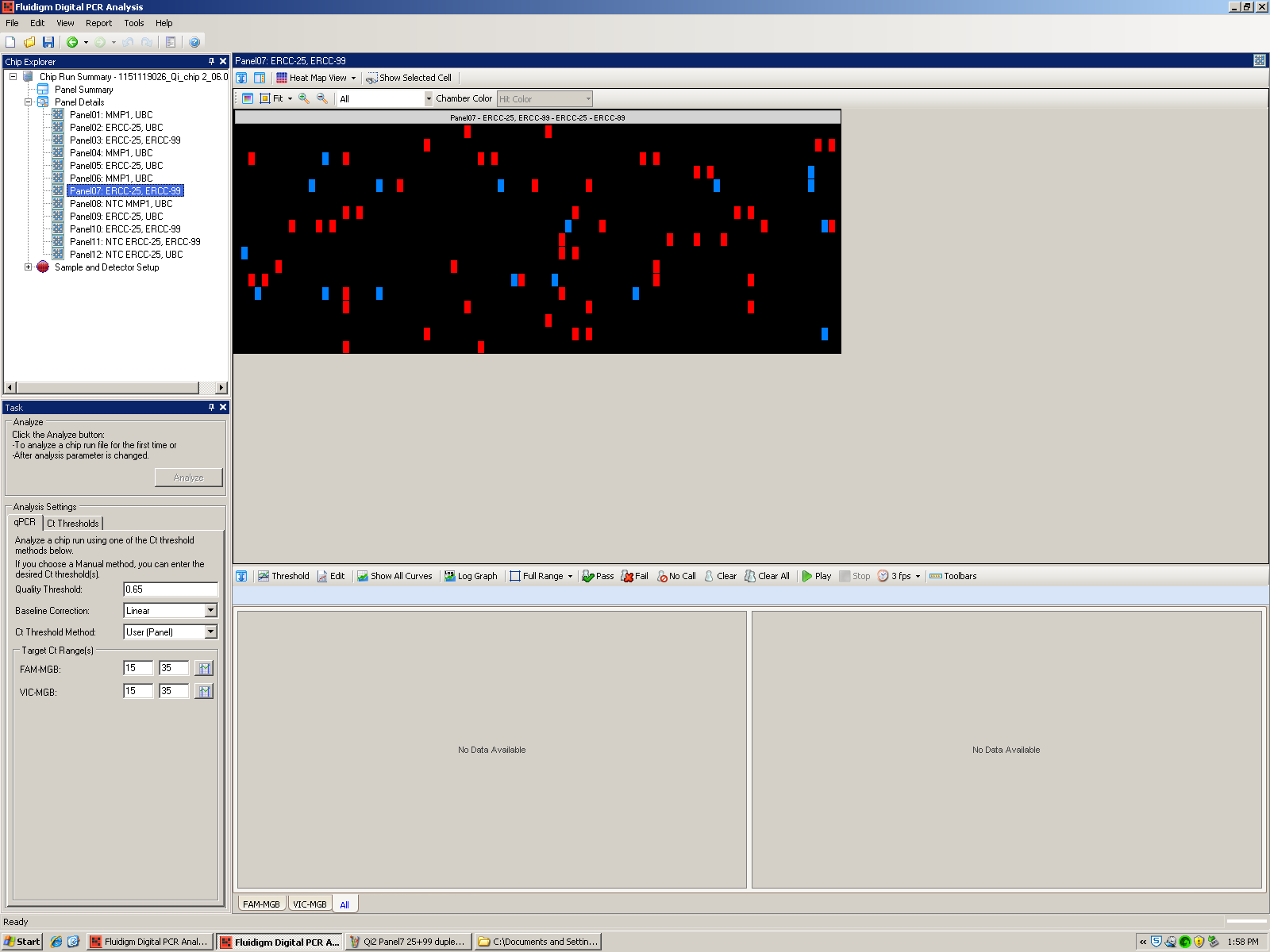

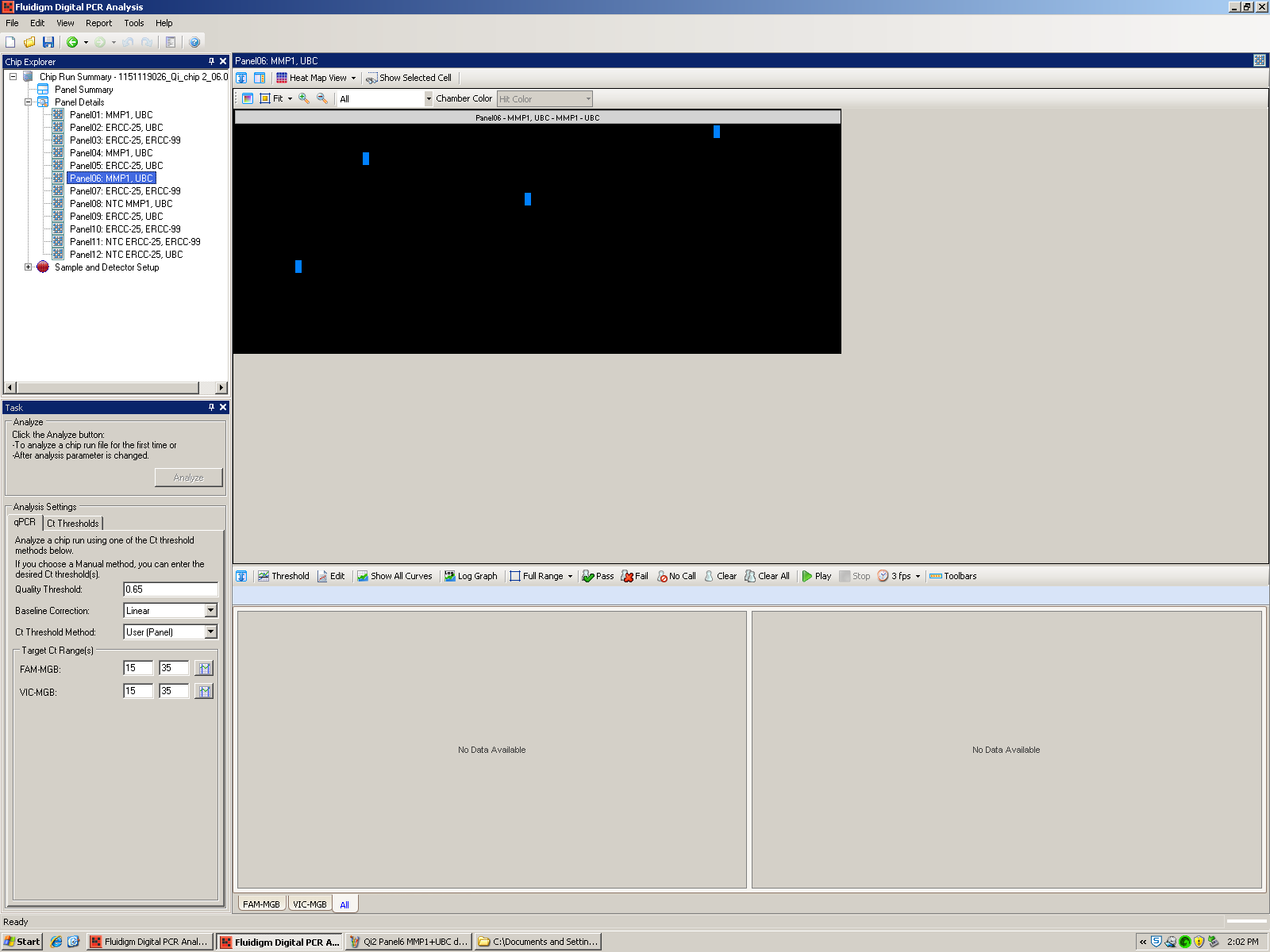

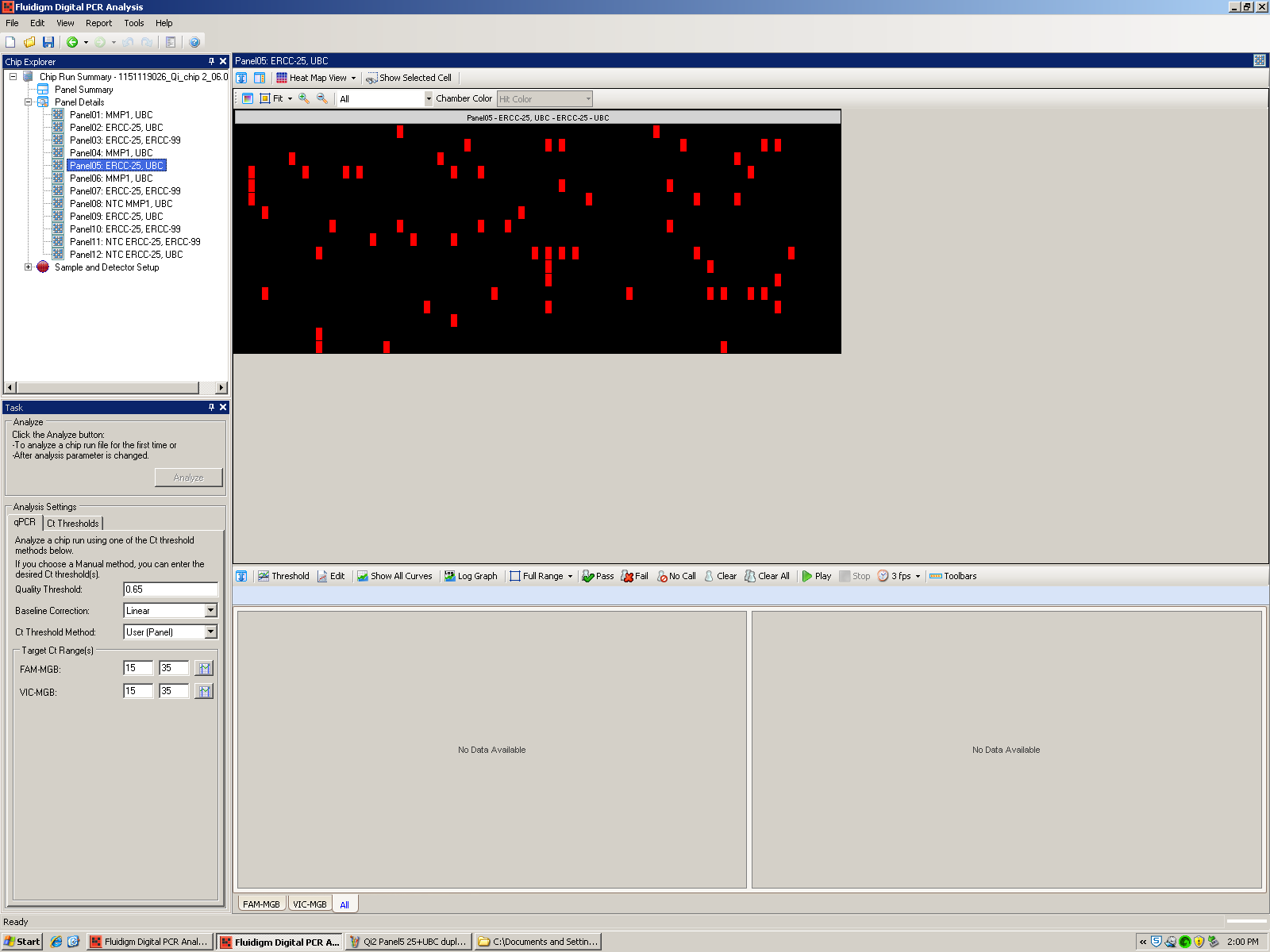

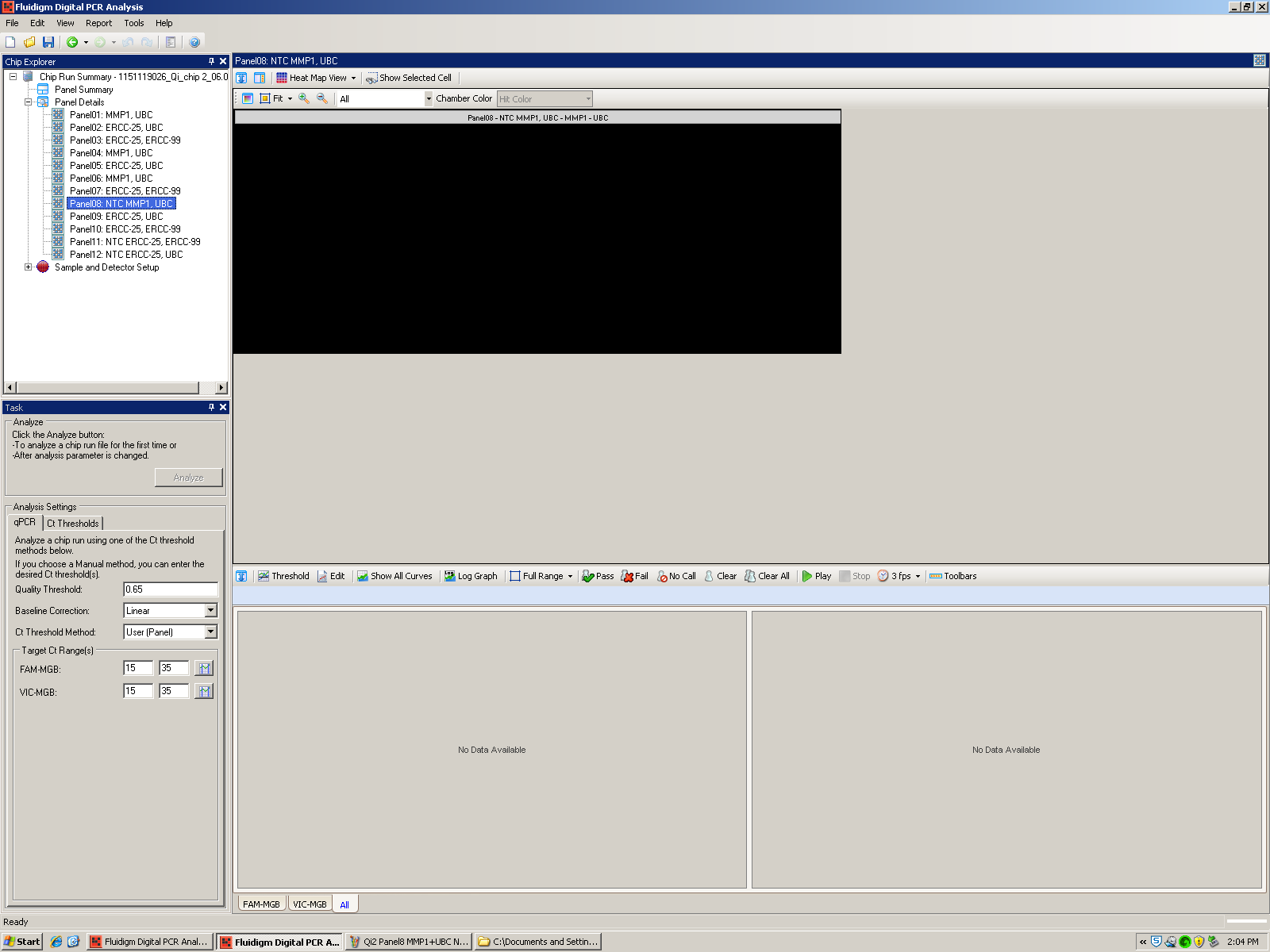

Supplement: Figure S3 — Typical dPCR output data from this study. Both amplification plots and heatmaps are shown. Amplification plots display ΔRN versus cycle number. Heatmaps are the corresponding schematic representations of positive partitions as detected by the Biomark instrument. Black = no amplification. Red = FAM amplification. Blue = HEX amplification. Threshold was adjusted to eliminate cross talk between the filters (FAM versus HEX). (A) One-Step RT-qPCR Kit Comparison by dPCR. (B) Endogenous versus Synthetic Targets. (DOCX) [file pone.0075296.s003.docx]
