# Supplementary material for: Evaluation of Digital PCR for Absolute RNA Quantification
Source: PLoS One. 2013 Sep 20;8(9):e75296. doi: 10.1371/journal.pone.0075296 (PMC3779174; doi:10.1371/journal.pone.0075296)
Supplement: Table S1 — Primer and probe sequences. (DOCX) [file pone.0075296.s004.docx]

**Table S1 Primer and probe sequences**

| **Target DNA** | **Gene Accession Number** | **Primer/Probe Sequence (5’ to 3’)** | |
| --- | --- | --- | --- |
| **Synthetic targets:** | | |  |
| ERCC-00013 | EF011062 | (F) CGGACATGGTGTTGGTCAAG  (R) TTGTTGGGCGGACCGTAA  (P) FAM-TGCATGAGGACCCGCAAATTCCTC-BHQ1 | |
| ERCC-00025 | DQ883689 | (F) CGGTCGTGAACTGCTATAGGA  (R) GGTAGTTTCGCTGGTTCGTT  (P) FAM-AGCCTGATACGAGCGCACAACA-BHQ1 | |
| ERCC-00042 | DQ516783 | (F) AGAGAGCTTTTGGCAATCCT  (R) TCATTTGCTAAGGCAGTTAAAGA  (P) FAM-TCACCAGTTCCCATGAATGTTCCAC-BHQ1 | |
| ERCC-00099 | DQ875387 | (F) TCGTCCATCCCTCAAGAGAGA  (R) CGCAATCGCGTGTGAATG  (P) FAM-CATGGAAAGAGCTCGACAAAATTTACTC-BHQ1 | |
| ERCC-00113 | DQ883663 | (F) GCGACACCAACATCGTTACG  (R) CCGCGCGTGAGCACTT  (P) FAM-ACACACCGGACGCTTGGATCAGTG-BHQ1 | |
| ERCC-00171 | DQ854994 | (F) TTAGTTTCGTGGCGGGATTT  (R) CACGAATCGCACGGATGTT  (P) FAM-AGGAAAACTGCGACTGTTCTTTAACC-BHQ1 | |
| **Endogenous Targets:** | | |  |
| MMP1 | NM_001145938.1 | (F) GGCCCACAAACCCCAAA  (R) TCTACCCGGAAGTTGAGCTCA  (P) FAM- AAGACAGATTCTACATGCGCA-MGB | |
| UBC | NM_021009.5 | (F) TTGTGGATCGCTGTGATCGT  (R) AGACTCTGACTGGTAAGACCATCACC  (P) FAM- ACTTGACAATGCAGATCT-MGB | |

(F) forward primer, (R) reverse primer, (P) probe, BHQ1: black hole quencher 1, MGB: minor groove binder. ERCC assays previously described by Devonshire et al. (2011) [[24](#_ENREF_24)]. RT-dPCR optimised assay concentrations: 600 nM (F), 400 nM (R), 180 nM (P).
