# Supplementary material for: Evaluation of Digital PCR for Absolute RNA Quantification
Source: PLoS One. 2013 Sep 20;8(9):e75296. doi: 10.1371/journal.pone.0075296 (PMC3779174; doi:10.1371/journal.pone.0075296)
Supplement: Table S2 — ERCC RNA concentration and copy number estimates. (DOCX) [file pone.0075296.s005.docx]

**Table S2 ERCC RNA concentration and copy number estimates**

| **ERCC-** | **Concentration (ng/µL)*** | **Molecular weight with poly(A) tail (g/mol)^†^** | **Estimated copy number/µL** | **Estimated copies/ng** |
| --- | --- | --- | --- | --- |
| 13 | 441.96 | 261410.3 | 1.02E+12 | 2.30E+09 |
| 25 | 427.88 | 640925.1 | 4.02E+11 | 9.40E+08 |
| 42 | 395.12 | 325738.6 | 7.30E+11 | 1.85E+09 |
| 99 | 400.97 | 434398.3 | 5.56E+11 | 1.39E+09 |
| 113 | 815.27 | 271727.2 | 1.81E+12 | 2.22E+09 |
| 171 | 384.65 | 163018.9 | 1.42E+12 | 3.69E+09 |

*As estimated by UV, n = 3. ^†^The inverse of the molecular weight (Mw) is the number of moles of template present in one gram of material. By multiplying the moles/gram by Avogadro's number, 6.023E+23 molecules/mole, the number of template molecules per gram can be calculated. The number of template molecules in the sample can then be estimated by multiplying copies/gram by 1.0E+09 to convert to ng and then multiplying by the amount of template (ng).
