# Supplementary material for: Evaluation of Digital PCR for Absolute RNA Quantification
Source: PLoS One. 2013 Sep 20;8(9):e75296. doi: 10.1371/journal.pone.0075296 (PMC3779174; doi:10.1371/journal.pone.0075296)
Supplement: Table S3 — Assay Positions. (DOCX) [file pone.0075296.s006.docx]

**Table S3 Assay Positions**

| **Target** | **Transcript Length (bases)*** | **Base position in transcript (5’ to 3’)** | | | | |
| --- | --- | --- | --- | --- | --- | --- |
|  |  | **Forward Primer** | **Probe** | | **Reverse Primer** | |
| MMP1 | 1903 | 802-818 | | 884-904 | | 914-934 |
| UBC | 2594 | 430-449 | | 451-468 | | 475-500 |
| ERCC-13 | 808 | 540-559 | | 563-586 | | 588-605 |
| ERCC-25 | 1994 | 1772-1792 | | 1795-1816 | | 1819-1838 |
| ERCC-42 | 1023 | 680-699 | | 703-727 | | 730-752 |
| ERCC-99 | 1350 | 732-752 | | 756-783 | | 785-802 |
| ERCC-113 | 843 | 80-99 | | 103-126 | | 129-144 |
| ERCC-171 | 505 | 237-256 | | 258-283 | | 285-303 |

*Length of transcript including poly(A) tail.
