# Supplementary material for: Evaluation of Digital PCR for Absolute RNA Quantification
Source: PLoS One. 2013 Sep 20;8(9):e75296. doi: 10.1371/journal.pone.0075296 (PMC3779174; doi:10.1371/journal.pone.0075296)
Supplement: Table S4 — MIQE checklist for authors, reviewers and editors. (DOCX) [file pone.0075296.s007.docx]

**Table S4 MIQE checklist for authors, reviewers and editors.**

| **ITEM TO CHECK** | **IMPORTANCE** | **CHECKLIST** | **COMMENTS/**  **WHERE?** |
| --- | --- | --- | --- |
| **EXPERIMENTAL DESIGN** | | | |
| Definition of experimental and control groups | **E** | **YES** | Materials and Methods |
| Number within each group | **E** | **YES** | Materials and Methods |
| Assay carried out by core lab or investigator's lab? | D | **YES** | Investigators lab |
| Acknowledgement of authors' contributions | D | **NO** | Not required by journal |
| **SAMPLE** | | | |
| Description | **E** | **N/A** |  |
| Volume/mass of sample processed | D | **N/A** |  |
| Microdissection or macrodissection | **E** | **N/A** |  |
| Processing procedure | **E** | **N/A** |  |
| If frozen - how and how quickly? | **E** | **N/A** |  |
| If fixed - with what, how quickly? | **E** | **N/A** |  |
| Sample storage conditions and duration (especially for FFPE samples) | **E** | **N/A** |  |
| **NUCLEIC ACID EXTRACTION** | | | |
| Procedure and/or instrumentation | **E** | **YES** | Materials and Methods |
| Name of kit and details of any modifications | **E** | **YES** | Materials and Methods |
| Source of additional reagents used | D | **YES** | Materials and Methods |
| Details of DNase or RNase treatment | **E** | **YES** | Materials and Methods |
| Contamination assessment (DNA or RNA) | **E** | **YES** | DN*ase* treatment – available on request |
| Nucleic acid quantification | **E** | **YES** | Materials and Methods |
| Instrument and method | **E** | **YES** | Materials and Methods |
| Purity (A260/A280) | D | **YES** | Available on request |
| Yield | D | **YES** | Available on request |
| RNA integrity method/instrument | **E** | **YES** | Materials and Methods |
| RIN/RQI or Cq of 3' and 5' transcripts | **E** | **YES** | Available on request |
| Electrophoresis traces | D | **YES** | Available on request |
| Inhibition testing (Cq dilutions, spike or other) | **E** | **YES** | Figure 3 |
| **REVERSE TRANSCRIPTION** | | | |
| Complete reaction conditions | **E** | **YES** | Materials and Methods |
| Amount of RNA and reaction volume | **E** | **YES** | Materials and Methods |
| Priming oligonucleotide (if using GSP) and concentration | **E** | **YES** | Materials and Methods |
| Reverse transcriptase and concentration | **E** | **YES** | Materials and Methods |
| Temperature and time | **E** | **YES** | Materials and Methods |
| Manufacturer of reagents and catalogue numbers | D | **YES** | Manufacturer: Materials and Methods |
| Cqs with and without RT | D* | **N/A** |  |
| Storage conditions of cDNA | D | **N/A** |  |
| **qPCR TARGET INFORMATION** | | | |
| Sequence accession number | **E** | **YES** | Supplementary Information |
| Location of amplicon | D | **YES** | Assay sequence: Supplementary Information |
| Amplicon length | **E** | **YES** | Assay sequence: Supplementary Information |
| *In silico* specificity screen (BLAST, etc) | **E** | **YES** | Available on request |
| Pseudogenes, retropseudogenes or other homologs? | D | **YES** | None detected by BLAST |
| Sequence alignment | D | **YES** | Available on request |
| Secondary structure analysis of amplicon | D | **YES** | Supplementary Information |
| Location of each primer by exon or intron (if applicable) | **E** | **YES** | Assay sequence: Supplementary Information |
| What splice variants are targeted? | **E** | **N/A** |  |
| **qPCR OLIGONUCLEOTIDES** | | | |
| Primer sequences | **E** | **YES** | Supplementary Information |
| RTPrimerDB Identification Number | D | **N/A** |  |
| Probe sequences | D** | **YES** | Supplementary Information |
| Location and identity of any modifications | **E** | **N/A** |  |
| Manufacturer of oligonucleotides | D | **YES** | Materials and Methods |
| Purification method | D | **YES** | HPLC |
| **qPCR PROTOCOL** | | | |
| Complete reaction conditions | **E** | **YES** | Materials and Methods |
| Reaction volume and amount of cDNA/DNA | **E** | **YES** | Materials and Methods |
| Primer, (probe), Mg++ and dNTP concentrations | **E** | **YES** | Materials and Methods; Manufactures proprietary |
| Polymerase identity and concentration | **E** | **YES** | Materials and Methods |
| Buffer/kit identity and manufacturer | **E** | **YES** | Materials and Methods |
| Exact chemical constitution of the buffer | D | **NO** | Manufactures proprietary |
| Additives (SYBR Green I, DMSO, etc.) | **E** | **N/A** |  |
| Manufacturer of plates/tubes and catalog number | D | **YES** | Lo Bind tubes (Eppendorf) |
| Complete thermocycling parameters | **E** | **YES** | Materials and Methods |
| Reaction setup (manual/robotic) | D | **YES** | Manual setup |
| Manufacturer of qPCR instrument | **E** | **YES** | Materials and Methods |
| **qPCR VALIDATION** | | | |
| Evidence of optimisation (from gradients) | D | **YES** | Available on request |
| Specificity (gel, sequence, melt, or digest) | **E** | **NO** | Not possible after digital PCR |
| For SYBR Green I, Cq of the NTC | **E** | **N/A** |  |
| Standard curves with slope and y-intercept | **E** | **N/A** | Digital PCR |
| PCR efficiency calculated from slope | **E** | **N/A** | Digital PCR |
| Confidence interval for PCR efficiency or standard error | D | **N/A** | Digital PCR |
| R2 of standard curve | **E** | **N/A** | Digital PCR |
| Linear dynamic range | **E** | **YES** | Results and Discussion |
| Cq variation at lower limit | **E** | **N/A** | Digital PCR |
| Confidence intervals throughout range | D | **YES** | Figure 3 |
| Evidence for limit of detection | **E** | **YES** | Figure 3 |
| If multiplex, efficiency and LOD of each assay. | **E** | **YES** | Figure 3 |
| **DATA ANALYSIS** | | | |
| qPCR analysis program (source, version) | **E** | **YES** | Materials and Methods |
| Cq method determination | **E** | **N/A** | Digital PCR |
| Outlier identification and disposition | **E** | **YES** | Materials and Methods |
| Results of NTCs | **E** | **YES** | Table 1 |
| Justification of number and choice of reference genes | **E** | **N/A** |  |
| Description of normalisation method | **E** | **N/A** |  |
| Number and concordance of biological replicates | D | **N/A** |  |
| Number and stage (RT or qPCR) of technical replicates | **E** | **YES** | Materials and Methods |
| Repeatability (intra-assay variation) | **E** | **YES** | Materials and Methods |
| Reproducibility (inter-assay variation, %CV) | D | **YES** | Results and Discussion |
| Power analysis | D | **No** | Figure 3 |
| Statistical methods for result significance | **E** | **YES** | Materials and Methods |
| Software (source, version) | **E** | **YES** | Materials and Methods |
| Cq or raw data submission using RDML | D | **N/A** |  |

All essential information (E) must be submitted with the manuscript. Desirable information (D) should be submitted if available. If using primers obtained from RTPrimerDB, information on qPCR target, oligonucleotides, protocols and validation is available from that source.

*: Assessing the absence of DNA using a no RT assay is essential when first extracting RNA. Once the sample has been validated as DNA-free, inclusion of a no-RT control is desirable, but no longer essential.

**: Disclosure of the probe sequence is highly desirable and strongly encouraged. However, since not all commercial pre-designed assay vendors provide this information, it cannot be an essential requirement. Use of such assays is advised against.
