# Supplementary material for: Evaluation of Digital PCR for Absolute RNA Quantification
Source: PLoS One. 2013 Sep 20;8(9):e75296. doi: 10.1371/journal.pone.0075296 (PMC3779174; doi:10.1371/journal.pone.0075296)
Supplement: Appendix S1 — Materials and Methods. (DOCX) [file pone.0075296.s008.docx]

**Appendix S1**

**Materials and Methods**

*Cell Lines* Subculturing and propagation was performed as per manufacturer’s instructions, with appropriate culture medium and serum additionally supplied from ATCC (Hep-G2: Eagle's Minimum Essential Medium, 10% fetal bovine serum. SaOS-2: McCoy's 5a Medium Modified, 15% fetal bovine serum. Hs 683: Dulbecco's Modified Eagle's Medium, 10% fetal bovine serum).

Prior to sub-culturing of cells in T-175 flasks (Corning, Amsterdam, The Netherlands), culture medium was aspirated from cell monolayer at approximately 90% confluency, which was then washed briefly in room temperature Hanks Balanced Salt Solution (HBSS; PAA Laboratories, Somerset, UK) to remove serum. Five mL Trypsin/EDTA (Sigma) solution was added to each flask and incubated at 37 ºC for 5 min. After incubation, cell detachment was monitored under a light microscope until all cells had detached. Adding an equal volume of the appropriate culture media subsequently quenched trypsin activity. Cells were pooled together and then re-seeded into fresh T-175 flasks containing 30 mL of the appropriate cell culture media, according to their splitting ratio. For propagating cells in culture, 100% of the media was replaced every second day.

*Total RNA Extraction from Cell Lines* TRIzol lysates were thawed (following storage at -80°C after collection) before adding chloroform (Sigma) (200 µL of chloroform for every 1 mL of TRIzol). Following phase separation using centrifugation (12,000 × g for 15 min at 4ºC), RNA was collected in the upper aqueous phase and precipitated using 0.5 mL isopropyl alcohol (Sigma) per 1 mL TRIzol. The RNA pellet was then washed with 75% ethanol (Sigma) before resuspension in 50 µL nuclease-free water (Life Technologies).
