# Supplementary material for: Evaluation of Digital PCR for Absolute RNA Quantification
Source: PLoS One. 2013 Sep 20;8(9):e75296. doi: 10.1371/journal.pone.0075296 (PMC3779174; doi:10.1371/journal.pone.0075296)
Supplement: Appendix S2 — dPCR Calculations Explained. (DOCX) [file pone.0075296.s009.docx]

**Appendix S2**

**dPCR Calculations Explained**

The number of partitions in the dPCR chips used is sufficiently large to use Poisson probabilistic analysis to broadly estimate the mean concentration of the RNA sample using the following Excel formula:

λ = -LN(1-(№ Positive partitions /C))

№ Copies on the panel = (LN((C – № positive partitions)/C))*(-C)

№ Positive partitions = C-((EXP(№ copies in the panel/-C))*C)

*Target copies/µL = (№ copies on the panel)/(proportion loaded per panel)

Proportion loaded per panel = (volume loaded per panel)/(volume loaded per inlet)

Volume loaded per panel = (Partition volume) X (total № partitions per panel)

*Where*:

λ = the number of molecules per partition

C = № partitions analysed per panel

№ Copies in the panel ≡ estimated copies

Inlet = Individual well for sample loading (discrete for each panel)

*Target copies/µL is the concentration of sample as it is added to master mix. If dilutions have been made to get to this point, these also need to be considered in your calculation of target copies/µL in your original sample.

|  | **12.765** | **48.770** |
| --- | --- | --- |
| № panels | 12 | 48 |
| C | 765 | 770 |
| Master reaction volume prepared per inlet | 8 µL | 4 µL |
| Volume of master reaction loaded per panel | 4.6 µL | 0.65 µL |
| Proportion loaded per panel | 0.575 | 0.1625 |
| Partition volume | 6 nL | 0.84 nL |

- So for example, for a sample with 500 positive partitions (amplifications), using a 48.770 dPCR chip:

№ Copies on the panel = (LN((770 – 500)/770))*(-770) = 806.9358

- Then:

Target copies/µL in dilution used = 806.9358/0.1625 = 4965.759

- Original sample was diluted 1:1000 for experiment. Therefore:

Original sample concentration = 4965.759 X 1000 = 4965759 copies/µL

= 4.97E+06 copies/µL

Lamda (**λ**), defined as the number of molecules per partition or true concentration, may be determined with the least amount of relative error when there are approximately 1.6 target molecules per partition, which corresponds to approximately 80% positive partitions, at the 95% confidence interval [[42](#_ENREF_42)]. That equates to approximately 612 or 616 positive partitions per panel and 1231 or 1239 copies/panel, for 12:765 or 48.770 dPCR chips respectively.
